# Potency Assessment of CBD Oils by Their Effects on Cell Signaling Pathways

**DOI:** 10.3390/nu12020357

**Published:** 2020-01-30

**Authors:** Yasuyo Urasaki, Cody Beaumont, Michelle Workman, Jeffery N. Talbot, David K. Hill, Thuc T. Le

**Affiliations:** 1 College of Pharmacy, Roseman University of Health Sciences, 10530 Discovery Drive, Las Vegas, NV 89135, USA; yurasaki@roseman.edu (Y.U.); jtalbot@roseman.edu (J.N.T.); 2 dōTERRA International, LLC, 389 South 1300 West, Pleasant Grove, UT 84062, USA; cbeaumont@doterra.com (C.B.); marndt@doterra.com (M.W.); drhill@doterra.com (D.K.H.)

**Keywords:** cannabidiol, protein posttranslational modification, nanofluidic proteomics, neuronal signaling pathways, adulteration, potency assessment, capillary isoelectric focusing

## Abstract

This study used nanofluidic protein posttranslational modification (PTM) profiling to measure the effects of six cannabidiol (CBD) oils and isolated CBD on the signaling pathways of a cultured SH-SY5Y neuronal cell line. Chemical composition analysis revealed that all CBD oils met the label claims and legal regulatory limit regarding the CBD and tetrahydrocannabinol (THC) contents, respectively. Isolated CBD was cytotoxic, with an effective concentration (EC_50_) of 40 µM. In contrast, the CBD oils had no effect on cell viability at CBD concentrations exceeding 1.2 mM. Interestingly, only an unadulterated CBD oil had strong and statistically significant suppressive effects on the pI3K/Akt/mTOR signaling pathway with an EC_50_ value of 143 µM and a slow-acting timescale requiring hours. Systematic profiling of twenty-six proteins, which served as biomarkers for nine signaling pathways, revealed that the unadulterated CBD oil downregulated seven signaling pathways but had no measurable effect on the other two signaling pathways. The remaining CBD oils, which were adulterated, and isolated CBD had weak, variable, or undetectable effects on neuronal signaling pathways. Our data clearly showed that adulteration diminished the biological activities of CBD oils. In addition, nanofluidic protein PTM profiling provided a robust means for potency assessment of CBD oils.

## 1. Introduction

The consumer demand for hemp-derived products is growing rapidly in the United States following a recent change in federal legislation and the approval of Epidiolex, an oral cannabidiol (CBD) solution, for the treatment of rare and severe forms of epilepsy by the Food and Drug Administration (FDA) [1]. The 2018 Farm Bill legalized hemp and removed its designation as a Schedule I controlled substance. Hemp is any part of the *Cannabis sativa* plant that contains less than 0.3% tetrahydrocannabidiol (THC) by weight, which includes the concentrated liquid extract known as CBD oil. However, the FDA continues to regulate CBD products. Any CBD product with a claim of therapeutic benefit must obtain FDA approval before it can be sold. In addition, the FDA prohibits the introduction of CBD into the food supply and dietary supplements because CBD is an active ingredient in an FDA-approved drug. Nonetheless, the prevalence of CBD oils as a wellness product continues to increase exponentially.

Despite the immense popularity of CBD oils, there is currently no industry-level regulation on their manufacturing process or quality standards [2]. Consequently, there are no reliable CBD selection guidelines or criteria that can help consumers identify high-quality and safe products. As a recreational product, CBD oils are loosely regulated by labeling accuracy of CBD content and compliance with the legal regulatory limit regarding THC content. In recent years, the FDA has been sending numerous warning letters to companies for inconsistent labeling of their products, where the THC content exceeds the regulatory limit or the CBD content is less than that labeled [3]. In addition, a scientific study of 84 CBD oils from 31 companies found that up to 69% of CBD oils evaluated were mislabeled [4]. Most worryingly, CBD oil adulteration, such as dilution, blending, and rectification are legally permissible. Adulteration of CBD oils most likely diminishes the benefits associated with the entourage effects of the natural constituents [5,6,7,8]. Furthermore, acute poisoning due to the addition of a synthetic cannabinoid to CBD products has been reported by a growing number of users [9]. Clearly, the CBD oil industry needs manufacturing and testing standards, regulatory oversight, and further research. 

In this study, a novel approach to potency assessment of CBD oils was examined. In addition to determining the chemical profiles of cannabinoids and terpenes, which are the chemical compounds that affect neurotransmission and give cannabis its distinctive smell, respectively, CBD oil potency was further evaluated by their effects on selected signaling pathways of an SH-SY5Y cell line. The SH-SY5Y cell line has been widely used as a neuronal cell model in Parkinson’s disease (PD) research due to the conservation of the genes and pathways associated with PD pathogenesis [10]. SH-SY5Y cells display a catecholaminergic phenotype and are capable of synthesizing both dopamine and noradrenaline. Due to the ease of their maintenance, SH-SY5Y cells can serve as a robust model to evaluate the biological effects of CBD oils on neuronal signaling pathways. Signaling pathways are cascades of protein kinases that sense and transmit external stimuli to elicit cellular responses. Different signaling pathways are responsive to different stimuli and regulate different cellular responses. For example, the pI3K/Akt/mTOR signaling pathway is responsive to glucose and regulates cell growth and metabolism, whereas the JAK/STAT signaling pathway is responsive to cytokines and regulates cell immunity. Measuring the effects of CBD oils on cell signaling pathways provides a robust means to assess their biological activities and potency.

Multiplexed nanofluidic protein posttranslational modification (PTM) profiling assays were used to measure the activities of nine signaling pathways in cultured SH-SY5Y neuronal cells following treatment with CBD oils. Previously, nanofluidic protein PTM profiling was used to identify aberrant signaling activities in tissue biopsies of nonalcoholic fatty liver disease and breast carcinoma [11,12,13]. Recently, nanofluidic protein PTM profiling was used to further differentiate copaiba essential oils with identical GC-MS chemical profiles [14]. Briefly, nanofluidic protein PTM profiling uses matrix-filled capillaries to separate proteins based on charge using capillary isoelectric focusing (cIEF) immunoassays or size using capillary Western immunoassays. Proteins are immobilized to the sidewalls by photo-induced crosslinking and, then, sequentially probed with primary antibodies and secondary antibodies conjugated with horseradish peroxidases. Following incubation with detection reagents, separated proteins are detected by the resulting chemiluminescence. Protein PTMs are measured by changes to isoelectric points or by binding to primary antibodies that recognize specific modification sites. Multiplexed assays using up to 96 individual capillaries in a single automated operation permit high-throughput protein PTM profiling. Here, multiplexed nanofluidic protein PTM profiling was deployed to assess the potency of CBD oils by measuring their effects on neuronal signaling pathways. 

## 2. Materials and Methods

### 2.1. Isolated CBD and CBD Oils

Cannabidiol (10 mg/mL of isolated CBD in methanol solution) was purchased from Cayman Chemical (cat. no. 90081, Ann Arbor, MI, USA). The CBD oils (V1-V6) were purchased from six different vendors directly from their websites. The names of the vendors were withheld due to the lack of consent for disclosure. Information on the CBD oils and their composition used in this study is listed in Table 1. 

### 2.2. Cannabinoid and Terpene Profiles of CBD Oils 

The chemical profiles of CBD oils (V1-V6) were analyzed at three independent laboratories at the Aromatic Plant Research Center (APRC, Lehi, UT, USA), Botanacor (BTNCR, Denver, CO, USA), and dōTERRA (DTRR, Pleasant Groves, UT, USA). At all three laboratories, the cannabinoid and terpene profiles were analyzed with high-performance lipid chromatography (HPLC) and gas chromatography coupled with mass spectrometry (GC-MS) methods, respectively. Laboratory reports of cannabinoid and terpene profiles of all six CBD oils are provided in the Supplementary Materials.

### 2.3. GC-MS Analysis of Terpene Profiles 

The terpene profiles of CBD oils were analyzed using a GC-MS method described previously [14]. In addition, GC-MS experimental parameters are listed on the laboratory reports of terpene profiles from APRC in the Supplementary Materials.

### 2.4. HPLC Analysis of Cannabinoid Profiles 

The cannabinoid profiles of CBD oils were analyzed using an Agilent 1290 Infinity II HPLC with a diode array detector (Agilent, Santa Clara, CA, USA). Sample preparation consisted of dissolving 1.0 ml of CBD oil into 10.0 mL methanol. Approximately 5 µL of sample was injected into a Kinetex C-18 4.6 mm × 150 mm × 2.6 µm column. Each injection was repeated three times. The mobile phases comprised 0.1% trifluoroacetic acid for mobile phase A and acetonitrile for mobile phase B. The samples were run using a gradient; initial A: 100%, 0.5 mL/min to 4 min; and 4.10 min A: 100%, 1.0 mL/min to 10 min, A: 97% to 15 min, and A: 75% hold until 17 min; and 17.5 min A: 100%, 0.5 mL/min. Data was processed using OpenLab software. Identification and quantification of compounds in samples were compared to the standard using retention time and area of absorbance peak.

### 2.5. Cell Line and Culturing Condition

The SH-SY5Y human neuroblastoma cell line was obtained from the American Type Tissue Collection (cat. no. CRL2266, ATCC, Manassas, VA, USA). The SH-SY5Y cells were cultured in a 1:1 mixture of ATCC-formulated Eagle’s minimum essential medium (cat. no. 302003) and F12 medium (cat. no. 30-2004, ATCC) supplemented with 10% fetal bovine serum (cat. no. SH30088.03, GE Healthcare Life Sciences, Pittsburgh, PA, USA). 

### 2.6. Treatment Condition

The SH-SY5Y cells were cultured to approximately 70% confluence prior to the replacement of the culturing medium. The new culturing medium contained isolated CBD or CBD oils at the desired final CBD concentrations. The claimed CBD concentrations were used for the dilution calculation. The incubation time in the new culturing medium was the same as the time after treatment. For the experiments using isolated CBD, the control groups were treated with culturing medium mixed with methanol at dilutions that matched those of the isolated CBD. For experiments using CBD oils, the control groups were treated with culturing medium alone.

### 2.7. Cell Proliferation and Cytotoxicity Assays

MTS and crystal violet assay kits were obtained from Promega (cat. no. G3581, Madison, WI, USA) and Fisher Scientific (cat. no. C581, Waltham, MA, USA) and performed according to manufacturers’ protocols. MTS signals were detected with a multimode microplate reader (Synergy 2, BioTek, Winooski, VT, USA). Cell density following crystal violet staining was examined with a standard cell culture microscope equipped with a digital camera. All MTS and crystal violet assays were performed in triplicate, and duplicate experiments were performed per treatment condition, producing six repeated measurements per treatment condition. 

### 2.8. Preparation of Cell Lysates

Approximately 1 million SH-SY5Y cells were incubated with 60 µL of lysis buffer (Bicine/CHAPS, cat. no. 040-764, Protein Simple, Santa Clara, CA, USA) on ice for 10 min, sonicated four times for a duration of 5 s, and rotated for 2 h, at 4 °C. Following centrifugation at 12,000 rpm in an Eppendorf 5430R microfuge for 20 min at 4 °C, the supernatants were collected as cell lysates. The total protein concentration in cell lysates was determined with Bradford assays and adjusted to a final concentration of 0.3 µg/µL with separation gradients for charge-based immunoassays or 0.4 µg/µL with denaturing buffers for size-based immunoassays. 

### 2.9. Antibodies and Biomarker Proteins

The primary and secondary antibodies are listed in Supplemental Table S1. The name and function of biomarker proteins are listed in Supplemental Table S2.

### 2.10. cIEF Immunoassays

Cell lysates were added to separation gradients that contained pI standards (Premix G2, pH 5 to 8 or pH 3 to 10, Protein Simple) and transferred into 384-well plates for cIEF immunoassays using the NanoPro 1000 system (Protein Simple). Approximately 400 nanoliters of cell lysate were loaded automatically into each capillary of the 96-capillary system. Following isoelectric focusing at 15 mW for 50 min, proteins were crosslinked to inner capillary walls using ultraviolet irradiation for 80 s. Primary and horseradish peroxidase-conjugated secondary antibodies were sequentially introduced into each capillary, with incubation times of 120 min and 60 min, respectively. After incubation with chemiluminescence detection reagents, the protein signal was detected with an average exposure time of 240 s. All cIEF immunoassays were performed in triplicate for each protein, and duplicate experiments were performed per treatment condition, producing six repeated measurements per protein. Hsp70 was used as a loading control for all cIEF immunoassays. In the NanoPro 1000 system, the loading control (Hsp70) and proteins of interest were detected with chemiluminescence in separate capillaries.

### 2.11. Capillary Western Immunoassays

Cell lysates were mixed with denaturing buffers (cat. no. PS-ST01EZ or PS-ST03EZ, Protein Simple) and denatured at 95 °C for 5 min and, then, transferred to assay plates containing either 12 to 230 kDa or 66 to 440 kDa Separation Modules (cat. no. SM-W004 or SM-W008, Protein Simple). Blocking reagents, wash buffers, primary antibodies, secondary antibodies, and chemiluminescence substrates were prepared and dispensed into the same assay plates. Assay plates were loaded into the Jess system (Protein Simple). Size-based protein separation and detection were performed automatically in the individual capillaries using the default protocols. Typically, the protein separation time was 30 min at 475 volts. Incubation with primary and secondary antibodies was sequential with an incubation time of 30 min per antibody. The Jess system is capable of simultaneous near infrared fluorescence and chemiluminescence detection of loading controls and proteins of interest, respectively, within the same capillaries. HSP60 and β-actin were used as loading controls. All capillary Western immunoassays were performed in triplicate for each protein, and duplicate experiments were performed per treatment condition, producing six repeated measurements per protein. 

### 2.12. Data Analysis

Assignment of pI values to protein phosphoisoforms was based on the literature and our own data [15,16,17,18,19,20,21,22,23,24,25,26,27]. Quantitation of protein expression and phosphorylation levels were performed using Compass software from Protein Simple. The protein expression or phosphorylation levels were adjusted with loading controls. The expression level of phosphorylation at a specific residue in capillary Western immunoassays was further adjusted with the expression level of total protein. 

### 2.13. Statistical Analysis

Quantitative data were presented as mean values ± standard deviations across six repeated measurements. Statistical significance was calculated with a Student’s t-test and thresholding at *p* ≤ 0.05 versus control. 

### 2.14. Sample Availability

All CBD oils used for the current study are available from the corresponding author on reasonable request.

## 3. Results

### 3.1. Physical Appearances and Chemical Profiles of Six Full-Spectrum CBD Oils 

**Figure 1:**
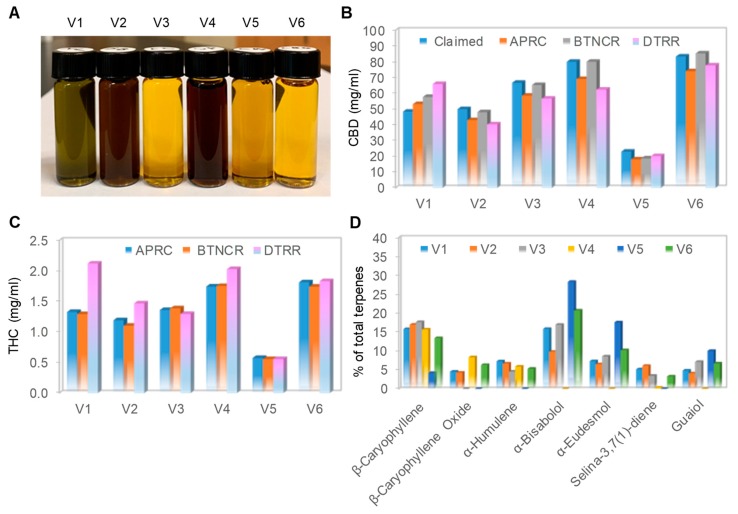
Physical and chemical profiles of CBD oils. (**A**) Physical appearance of six cannabidiol (CBD) oils captured with a standard digital camera; (**B**) claimed and measured CBD concentrations in the six CBD oils. Claimed CBD concentration (blue) and measured CBD concentrations by Aromatic Plant Research Center (APRC, orange), Botanacor (BTNCR, gray), and dōTERRA (DTRR, pink); (**C**) measured tetrahydrocannabinol (THC) concentration in the six CBD oils. Measured THC concentrations by APRC (blue), BTNCR (orange), and DTRR (pink). CBD and THC concentrations were independently measured by HPLC methods at three different laboratories: APRC, BTNCR, and DTRR; (**D**) major aroma compounds as a percentage of total terpenes in the six CBD oils. The terpene profiles were measured by a GC-MS method at APRC. CBD oil V1 (blue), V2 (orange), V3 (gray), V4 (yellow), V5 (blue), and V6 (green).

We purchased six full-spectrum CBD oils from the open market and subjected them to both physical appearance examination and chemical composition analysis. Full-spectrum CBD oils were extracted from whole hemp plants and contained cannabinoids, terpenes, and other natural compounds. According to the manufacturers, the CBD oils were extracted with either CO_2_ extraction methods (V1-V3) or ethanol extraction methods (V4-V6) (Table 1). CBD oil V1 was not adulterated with either dilution in carrier oil or addition of isolated CBD. In contrast, the remaining five CBD oils were adulterated with dilution in carrier oils; CBD oils V2 and V4 were diluted in hemp seed oils, V3 and V6 were diluted in medium-chain triglyceride oils, and V5 was diluted in olive oil. Furthermore, CBD oils V2-V6 were adulterated with the addition of isolated CBD. Interestingly, the CBD oils had very different colors when examined in clear glass vials (Figure 1A). Unadulterated CBD oil V1 had a distinctive greenish color. CBD oils V2 and V4, which were diluted in hemp seed oils, had a dark-brown color. CBD oils V3 and V6, which were diluted in medium-chain triglyceride oils, had a light yellow color. CBD oil V5, which was diluted in olive oil, had a dark yellow color. It appeared that the similar colors of CBD oils extracted with different methods (V2 versus V4 or V3 versus V6) were due to similar carrier oils. 

Independent laboratory analyses revealed that all six CBD oils had CBD content consistent with that listed on the labels, as the claimed and average measured CBD concentrations differed by less than 10% (Figure 1B). In addition, the measured THC content of all six CBD oils was compliant with the legal regulatory limit of less than 0.3% (Figure 1C). The measured terpene content of CBD oil V1 was the highest as compared with that of the remaining CBD oils, which was consistent with the claim by the manufacturer that V1 was not diluted in a carrier oil (Table 1). Within the volatile terpene fraction, β-caryophyllene was detected in all CBD oils (Figure 1D). The other terpenes were present in some CBD oils and absent in the others, with no discernable pattern. The terpene profiles confirmed that all six samples were full-spectrum CBD oils. By the current testing standards and metrics, all six CBD oils purchased had accurate labeling of CBD content and were complaint with the legal regulatory limit regarding THC content. 

### 3.2. Cytotoxicity of Isolated CBD to Cultured Neuronal Cells

**Figure 2:**
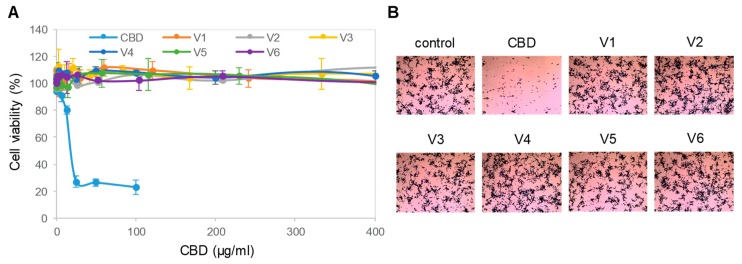
Toxicity of isolated CBD on neuronal cells. (**A**) Viability of SH-SY5Y cells as a function of treatment with isolated CBD and CBD oils. Error bars were standard deviations across 6 repeated measurements per experimental condition. Cell viability was measured with MTS assays; (**B**) density of SH-SY5Y cells as a function of treatment with a CBD isolate and CBD oils. SH-SY5Y cells were stained with crystal violet dye and examined with a standard brightfield cell culture microscope. The image field of view has a xy dimension of 1500 µm x 1125 µm.

Next, we evaluated the effects of isolated CBD and all six CBD oils on the viability of SH-SY5Y cells in culture. The SH-SY5Y cells were cultured to approximately 70% confluence and, then, treated with titrating concentrations of isolated CBD or CBD oils for 24 h. Cell viability was assessed with a colorimetric MTS assay that measured cell metabolic activity. We found that isolated CBD was highly cytotoxic to SH-SY5Y cells, with an effective EC_50_ concentration of approximately 12.5 µg/mL or 40 µM (Figure 2A). In contrast, treatment with the CBD oils at CBD concentrations up to 400 µg/mL or 1.27 mM had no effect on the viability of SH-SY5Y cells. The MTS data were further corroborated by a crystal violet assay, which stained remaining viable and adherent cells with a histological dye. Observation with brightfield microscopy revealed that isolated CBD dramatically reduced the density of SH-SY5Y cells compared with that of control cells (Figure 2B). At the same CBD concentration of 100 µg/mL or 318 µM, CBD oils had no effect on the density of SH-SY5Y cells as compared with that of the control cells. Therefore, CBD in the CBD oils did not have the same level of cytotoxicity as isolated CBD. 

### 3.3. Downregulation of the PI3K/Akt/mTOR Signaling Pathway

**Figure 3:**
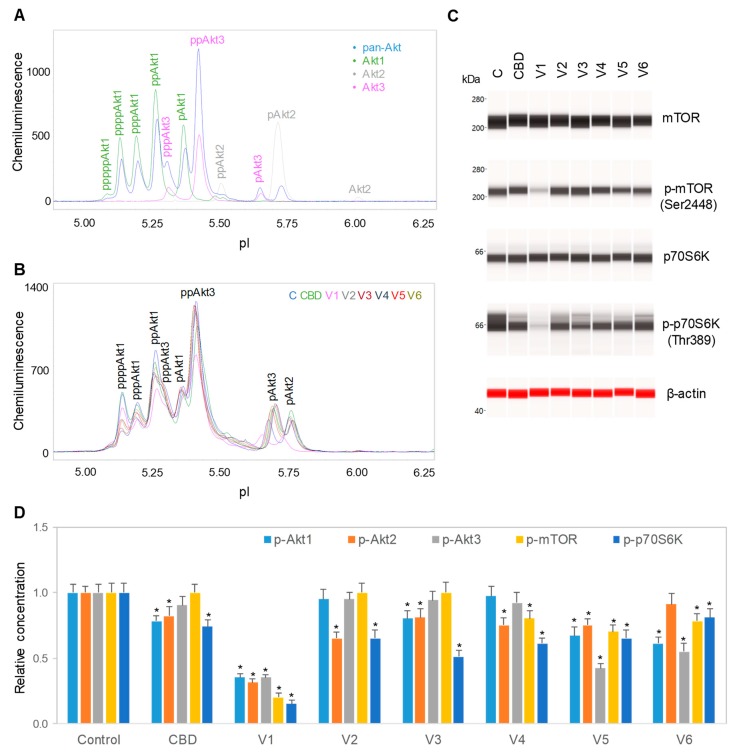
Downregulation of the PI3K/Akt/mTOR signaling pathway by isolated CBD and CBD oils. (**A**) AKT isoform identification in SH-SY5Y cell lysates using capillary isoelectric focusing (cIEF) immunoassays. Antibodies against total Akt (pan-Akt, blue), Akt1 (green), Akt2 (gray), or Akt3 (pink) were used to identify Akt isoforms; (**B**) the total Akt profiles of SH-SY5Y cells before (C, blue) and after 24 h of treatment with isolated CBD (green) or with various CBD oil samples, V1 (pink), V2 (gray), V3 (deep red), V4 (dark blue), V5 (red), and V6 (dark yellow); (**C**) capillary Western immunoassays using antibodies against mTOR, p-mTOR (Ser2448), p70S6K, p-p70S6K (Thr389), and β-actin (loading control). Values on the left side of the panels are molecular weights in kilodaltons (kDa); (**D**) the relative expression levels of the phosphoisoforms of Akt1 (p-Akt1, light blue), Akt2 (p-Akt2, orange), Akt3 (p-Akt3, gray), mTOR (p-mTOR (Ser2448), yellow), and p70S6K (p-p70S6K (Thr389), blue) before (control) or after 24 h of treatment with isolated CBD or CBD oil samples V1-V6. Relative concentration describes fold change of protein phosphoisoforms after treatment as compared with the control. Error bars are the standard deviations of six repeated measurements per experimental condition. Asterisks denote statistical significance for *p* < 0.05 versus controls. SH-SY5Y cells were treated with 6.25 µg/mL isolated CBD or CBD oils at 100 µg/mL final CBD concentration.

We further evaluated the effects of isolated CBD and CBD oils on the signaling activity of the pI3K/Akt/mTOR pathway, which is critical for the regulation of neuronal cell growth, longevity, and energy metabolism [28,29]. The expression levels of the phosphoisoforms of Akt, mTOR, and p70S6K were used to monitor the activity of the PI3K/Akt/mTOR signaling pathway. To minimize cytotoxicity, SH-SY5Y cells were treated with 6.25 µg/mL isolated CBD or 100 µg/mL CBD oils for 24 h prior to the collection of cell lysates for analysis. Akt phosphoisoforms were detected with cIEF immunoassays using primary antibodies against Akt1 isoforms, Akt2 isoforms, Akt3 isoforms, pan-Akt, or all Akt isoforms. Peak assignment of Akt isoforms on the electropherograms was based on the experimental data herein (Figure 3A), as well as on previously published data from multiple independent research groups [15,17]. In SH-SY5Y cells, the Akt3 isoforms were the dominant isoforms, accounting for 50%, followed by Akt1 isoforms at 40% and Akt2 isoforms at 10% (Figure 3A). Treatment of SH-SY5Y cells with isolated CBD and CBD oils generally reduced the expression of Akt phosphoisoforms, with the strongest suppressive effects induced by CBD oil V1 (Figure 3B and Supplemental Figures S1 and S2). Treatment of SH-SY5Y cells with CBD oil V1 strongly reduced the phosphorylation of mTOR at residue Ser2448 and of p70S6K at residue Thr389, which occur downstream of Akt, as detected with capillary Western immunoassays (Figure 3C). In contrast, isolated CBD and other CBD oils had either undetectable or weak suppressive effects on mTOR and p70S6K phosphorylation. Fold changes of protein phosphoisoforms after treatment, as compared with the control, were calculated to the yield the relative concentrations of individual signaling proteins. Quantitative analysis data further revealed strong, consistent, and statistically significant effects of CBD oil V1 on the reduction in the protein phosphoisoforms in the PI3K/Akt/mTOR signaling cascade (Figure 3D).

### 3.4. Dose- and Time-dependent Suppression of the PI3K/Akt/mTOR Signaling Pathway

**Figure 4:**
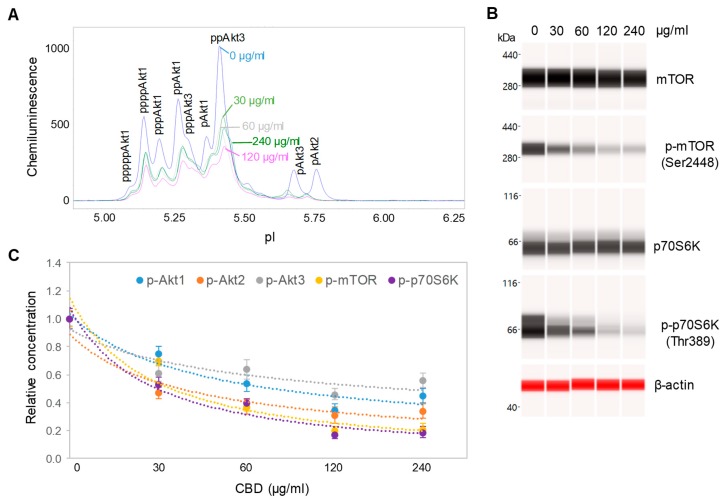
Dose-dependent negative regulation of the pI3K/Akt/mTOR signaling pathway by a CBD oil. (**A**) The pan-Akt profiles of SH-SY5Y cells as a function of CBD oil V1 dosages; (**B**) the expression levels of mTOR and p70S6K phosphoisoforms as a function of CBD oil V1 dosages; (**C**) the relative concentrations of Akt, mTOR, and p70S6K phosphoisoforms as a function of CBD oil V1 dosages. Relative concentration describes fold change of protein phosphoisoforms after treatment as compared with the control. Error bars are the standard deviations of six repeated measurements per experimental condition. SH-SY5Y cells were treated with various dosages of CBD oil V1 for 24 h.

**Figure 5:**
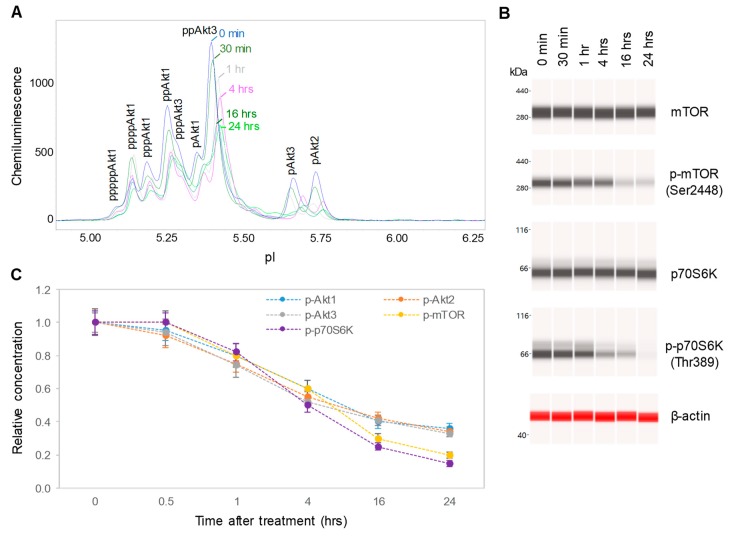
Time-dependent negative regulation of the pI3K/Akt/mTOR signaling pathway by a CBD oil. (**A**) The pan-Akt profiles of SH-SY5Y cells as a function of time after treatment with CBD oil V1; (**B**) the expression levels of mTOR and p70S6K phosphoisoforms as a function of time after treatment with CBD oil V1; (**C**) the relative concentrations of Akt, mTOR, and p70S6K phosphoisoforms as a function of time after treatment with CBD oil V1. Relative concentration describes fold change of protein phosphoisoforms after treatment as compared with the control. Error bars are the standard deviations of six repeated measurements per experimental condition. SH-SY5Y cells were treated with CBD oil V1 at 100 µg/mL final CBD concentration.

CBD oil V1 exhibited both dose- and time-dependent suppression of the PI3K/Akt/mTOR signaling pathway. Following treatment of SH-SY5Y cells with serial dilutions of CBD oil V1 for 24 h, we observed the suppression of phosphoisoforms of Akt1, Akt2, Akt3, mTOR, and p70S6K with an effective CBD concentration (EC_50_) of approximately 45 µg/mL or 143 µM (Figure 4A–C and Supplemental Figure S3). However, time-dependent measurements following the treatment of SH-SY5Y cells with 100 µg/mL CBD oil V1 revealed a slow-acting mechanism, in which 4 h were required to reach 50% suppression and 24 h to reach maximal suppression (Figure 5A–C and Supplemental Figure S4). Therefore, CBD oil V1 exerted negative regulatory control of the PI3K/Akt/mTOR signaling pathway.

### 3.5. Negative Regulation of Neuronal Signaling Pathways

**Figure 6:**
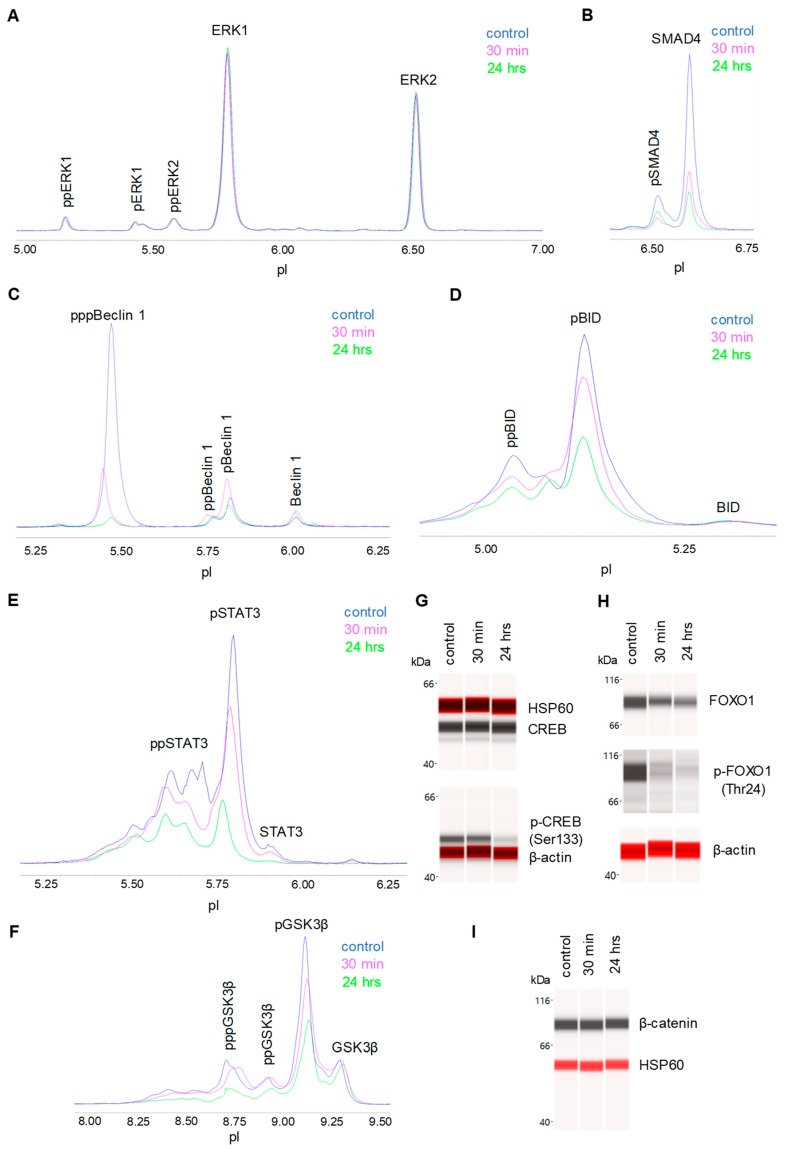
Proteomic profiling of the effects of a CBD oil. The proteomic profiles before and after 30 min and 24 h of treatment with CBD oil V1. (**A**) ERK1/2; (**B**) SMAD4; (**C**) beclin-1; (**D**) BID; (**E**) STAT3; (**F**) GSK3β; (**G**) CREB; (**H**) FOXO1; and (**I**) β-catenin. SH-SY5Y cells were treated with CBD oil V1 at 100 µg/mL final CBD concentration.

**Figure 7:**
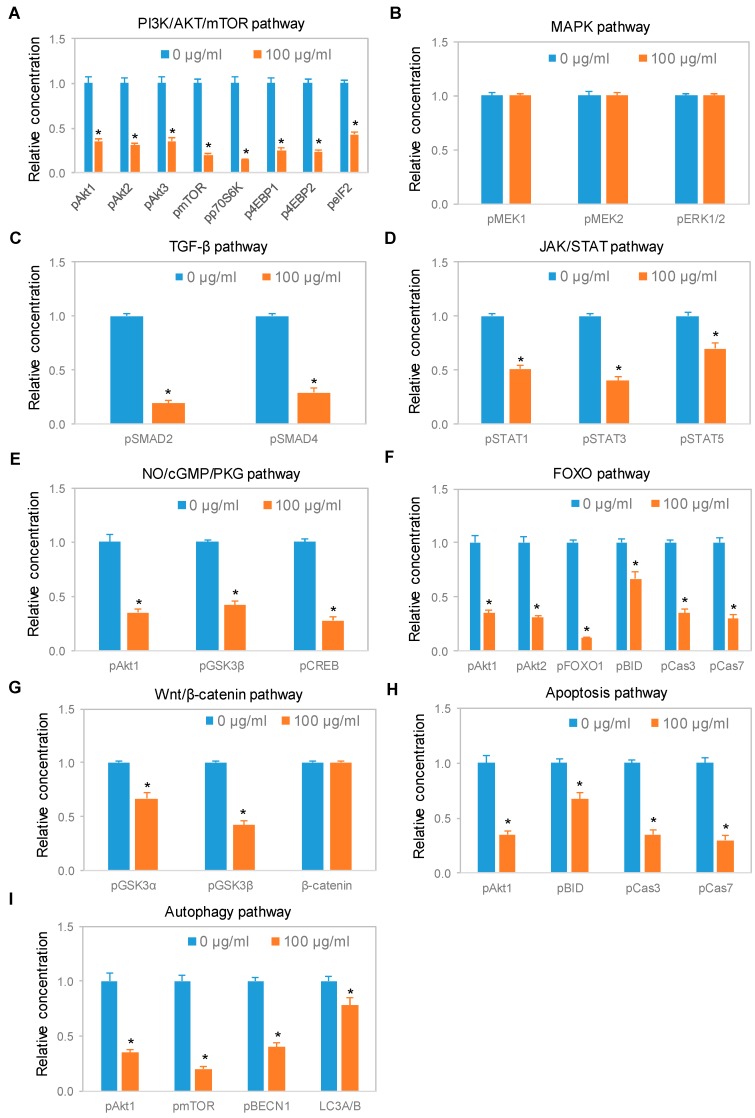
Expression of protein isoforms in selected signaling pathways following treatment with a CBD oil. (**A**) PI3K/Akt/mTOR pathway; (**B**) MAPK pathway; (**C**) TGF-β pathway; (**D**) JAK/STAT pathway; (**E**) NO/cGMP/PKG pathway; (**F**) FOXO pathway; (**G**) Wnt/β-catenin pathway; (**H**) apoptosis pathway; and (**I**) autophagy pathway. Blue and orange are control samples (0 µg/mL) and samples treated with CBD oil V1 at 100 µg/mL final CBD concentration for 24 h, respectively. Relative concentration describes fold change of protein phosphoisoforms after treatment as compared with the control. Error bars are standard deviations across six repeated measurements per experimental condition. Asterisks indicate statistical significance for *p* ≤ 0.05 versus control.

**Figure 8:**
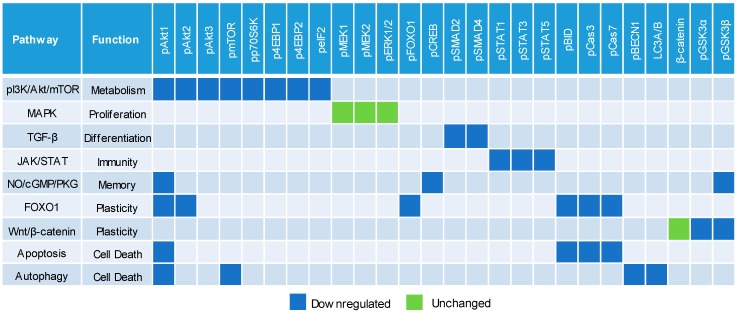
Downregulation of multiple cell signaling pathways by a CBD oil. Blue and green squares indicate downregulated and unchanged protein profiles, respectively, at 24 h post-treatment with CBD oil V1 at 100 µg/mL final CBD concentration. Squares in other colors indicate proteins that are not applicable to the given pathways.

We systematically evaluated the effects of CBD oil V1 on nine signaling pathways using a diagnostic panel of 26 proteins. Following the treatment of SH-SY5Y cells with CBD oil V1 at 100 µg/mL for 24 h, multiplexed cIEF, and Western immunoassays were performed on cell lysates. Representative data on selected proteins are presented in Figure 6A–I and Supplemental Figure S5A–L. Quantitative analysis with grouping of proteins into their respective signaling pathways is presented in Figure 7A–I. An alternative reporting format in table form is also presented in Figure 8. On the one hand, we found that CBD oil V1 suppressed the expression and phosphorylation of proteins in seven signaling pathways, namely, the PI3K/Akt/mTOR, TGF-β, JAK/STAT, NO/cGMP/PKG, FOXO, apoptosis, and autophagy pathways. On the other hand, CBD oil V1 had no effect on the expression or phosphorylation level of the proteins of the MAPK pathway or the expression level of β-catenin of the Wnt/β-catenin signaling pathway. Notably, isolated CBD and other selected CBD oils also exerted negative regulatory control of neuronal signaling pathways although the suppressive effects were much weaker than those observed with CBD oil V1 (Supplemental Figure S6A–L). 

## 4. Discussion

In this study, we reported differential effects on the viability and signaling activities of SH-SY5Y cells induced by isolated CBD and CBD oils. We found that isolated CBD had a negative effect on the viability of SH-SY5Y cells, with an EC_50_ value of approximately 40 µM. This observation was consistent with the reported cytotoxicity of isolated CBD and its proposed use as an antineoplastic agent in the literature [30,31]. Interestingly, CBD oils with CBD concentrations greater than 30 times that of isolated CBD had no measurable effect on the viability of SH-SY5Y cells. All six CBD oils purchased on the open market had their measured CBD concentrations match those of the label claims and THC concentrations below the legal regulatory limit. Surprisingly, CBD oil V1 exhibited the strongest, most consistent, and most statistically significant effects on multiple neuronal signaling pathways as compared with those produced by the remaining CBD oils. The weak effects of isolated CBD on neuronal signaling activities were most likely due to a low final concentration, which was intentionally used to avoid cytotoxicity. On the one hand, the final concentration of isolated CBD was 20 µM, which was seven times lower than the EC_50_ value of 143 µM for CBD oils. On the other hand, all CBD oils were used at a final concentration of 318 µM, which was at least two-fold greater than the EC_50_ value. However, the CBD oils exhibited differential effects on neuronal signaling activities. It would be convenient to cite the “entourage effects” or lack of these effects as the cause for the cytotoxicity of isolated CBD or weaker effects for diluted CBD oils versus those for undiluted CBD oils. However, the collected data were insufficient to prove or disprove the existence of such effects. Coincidently, the only unadulterated CBD oil among our samples exhibited the strongest suppressive effects on multiple neuronal signaling pathways. The remaining CBD oils, which had been adulterated, exhibited either undetectable or weak suppressive effects on neuronal signaling pathways. Therefore, CBD alone was an insufficient determinant of potency for CBD oils. 

Notably, nanofluidic protein PTM profiling proved to be a convenient and rapid means to assess the effects of CBD oils on neuronal signaling pathways. The PTM profiles of multiple proteins within a signaling cascade served as read-outs for CBD oil signaling activity and the associated functions. By measuring the expression and phosphorylation of a diagnostic panel of 26 proteins, we were able to assess the effects of CBD oils on the activities of nine signaling pathways critical for the regulation of neuronal functions. We reported that CBD oils downregulated seven signaling pathways that participate in metabolism, differentiation, immunity, memory, and cell death but had no effect on the other two pathways that regulate proliferation and synaptic plasticity. The suppressive effects of CBD oils on neuronal signaling pathways were slow acting, with a timescale of hours, and reached maximal suppression at 24 h post-treatment. The biological effects of CBD oils presented in this study could provide insights into the mechanisms underlying their purported benefits on relieving pain and anxiety [32]. Most significantly, nanofluidic protein PTM profiling provided a biochemical approach to differentiate CBD oils. It is plausible that the effects on cell signaling pathways could serve as a complementary determinant of potency for CBD oils.

The association of the endocannabinoid system with many neurodegenerative diseases renders this system a promising therapeutic target [33]. However, the therapeutic significance of cannabinoids is hindered by the intoxicating effects of THC, the principal psychoactive constituent of cannabis [34]. Alternatively, CBD is gaining popularity for medical uses due to its different mechanism of action from that of THC and its safety profile [2]. Current cannabinoid drug development is characterized by two distinctive paths with one path focused on the direct use of CBD oils and the other path focused on the use of natural or synthetic CBD [8]. On the one hand, the pursuit of purified CBD for drug development follows established guidelines for drug discovery with regard to quality, safety, and efficacy. On the other hand, the use of CBD oils for drug development faces seemingly insurmountable challenges with regard to established guidelines. Multiple constituents in CBD oils pose challenges to achieving manufacturing precision and evaluating pharmacology, toxicology, and efficacy in both preclinical and clinical studies. Nonetheless, CBD oils remain a promising candidate for drug development due to the potential synergy among the constituents [35], reduced adverse effects as compared with those of isolated CBD [36], and the usage history by millions of people to alleviate various medical conditions [37]. 

The potency assessment of CBD oils remains a significant challenge due to the lack of quality control standards. Current assessment of the quality of CBD oils has focused mainly on the chemical composition of cannabinoids and terpenes [38,39,40]. However, quality testing of CBD oils should include the assessment of their biological activities to ensure safety and potency. The observation that CBD by itself was an insufficient indicator of potency is consistent with several previous studies of essential oils, where it was found that not all biological activities were related to the main components [14,41,42]. Similar to the entourage effects in CBD oils, synergies among the constituents of essential oils or botanical extracts are critical for their biological activities [42,43,44,45]. Adulteration has been found to alter the biological activities of essential oils, as reported previously [14,41], and of CBD oils, as reported in this study. Being commercially marketed as a recreational product, CBD oils are often adulterated with dilution, blending, and rectification to enhance smell, taste, and appearance. However, consumers often use CBD oils as a wellness product for various medical conditions [2]. Adulterated CBD oils could pose a serious public health threat due to their altered biological activities. Recent cases of acute and severe respiratory distress related to vaping of CBD products together with THC and nicotine products [46,47,48,49,50,51] further highlight the need to understand the toxicity profiles of adulterated CBD oils. For medicinal applications, it is advisable to use pure and unadulterated CBD oils that have been tested for both chemical composition and biological activities. 

Signaling pathway analysis is an effective approach to understand the molecular mechanisms underlying the synergistic, potentiation, and antagonistic effects of the constituents of CBD oils. In this study, we showed that multiplexed immunoassays in nanocapillaries permitted simultaneous analysis of nine signaling pathways in cultured SH-SY5Y cells. Notably, nanofluidic protein PTM profiling is ideally suited for applications on clinical specimens due to its ultrasensitivity, where nanograms of tissue lysates are sufficient for the detection of picograms of proteins of interest [12,13,21,52]. Furthermore, high reproducibility between measurements, reliable quantitation, and low user errors due to automated operation are among the attributes that highlight the robustness of the technology [25,53]. Future applications of nanofluidic protein PTM profiling to characterize the effects of individual constituents of CBD oils or their combinations on cellular signaling pathways would assist in the rational designs of cannabinoid drugs. Most significantly, the versatility of nanofluidic protein PTM profiling would be invaluable for the assessment of the quality, safety, and efficacy of CBD oils in both preclinical and clinical studies.

## Figures and Tables

**Table 1:** Full-spectrum CBD oils.

CBD Oil	Extraction Method	Dilution	Addition of Isolated CBD	Measured Density (mg/mL)	Claimed CBD (mg/mL)	Measured CBD (mg/mL)	Measured THC (mg/mL)	Measured Terpenes (mg/mL)
V1	CO_2_	No	No	921.10	48.33	53.16–65.86	1.29–2.11	2.27
V2	CO_2_	HSO	Yes	915.50	50.00	40.28–48.05	1.10–1.46	1.49
V3	CO_2_	MCT	Yes	922.80	66.67	56.66–65.24	1.29–1.38	1.04
V4	ETHANOL	HSO	Yes	921.10	80.00	62.36–80.03	1.74–2.03	1.56
V5	ETHANOL	OO	Yes	922.70	23.00	18.09–20.21	0.55–0.57	0.17
V6	ETHANOL	MCT	Yes	915.10	83.33	74.02–85.29	1.74–1.83	1.56

* HSO: hemp seed oil; MCT: medium chain triglyceride; OO: olive oil.

## Supplementary Materials

The following are available online at https://www.mdpi.com/2072-6643/12/2/357/s1, Supplemental Figure S1: Measuring the effects of isolated CBD and CBD oils on the expression and phosphorylation of Akt isoforms, Supplemental Figure S2: Loading controls for cIEF immunoassays, Supplemental Figure S3: Dose-dependent effects of CBD oil V1 on the expression and phosphorylation of Akt isoforms, Supplemental Figure S4: Time-dependent effects of CBD oil V1 on the expression and phosphorylation of Akt isoforms, Supplemental Figure S5: Proteomic profiling of the effects of CBD oil V1 with multiplexed cIEF immunoassays, Supplemental Figure S6: Expression of protein isoforms in selected signaling pathways following treatment with isolated CBD and CBD oil V4, Supplemental Table S1: List of primary and secondary antibodies, Supplemental Table S2: List of biomarker proteins and their functions and supplemental data on the chemical composition of the CBD oils.
