# Supplementary material for: Potency Assessment of CBD Oils by Their Effects on Cell Signaling Pathways
Source: Nutrients. 2020 Jan 30;12(2):357. doi: 10.3390/nu12020357 (PMC7071207; doi:10.3390/nu12020357)
Supplement: Supplementary file 1 [file nutrients-12-00357-s001.pdf]

Supplementary Materials

Potency Assessment of CBD Oils by Their Effects on Cell Signaling Pathways

Yasuyo Urasaki[1], Cody Beaumont[2], Michelle Workman[2], Jeffery N. Talbot[1], David K. Hill[2], Thuc T. Le[1,*]

[1]College of Pharmacy, Roseman University of Health Sciences, 10530 Discovery Drive, Las Vegas, NV 89135, USA.

[2]dōTERRA International, LLC, 389 South 1300 West, Pleasant Grove, Utah 84062, USA.

*To whom correspondence should be addressed: Email: tle5@roseman.edu; Tel: 1-702-802-2820

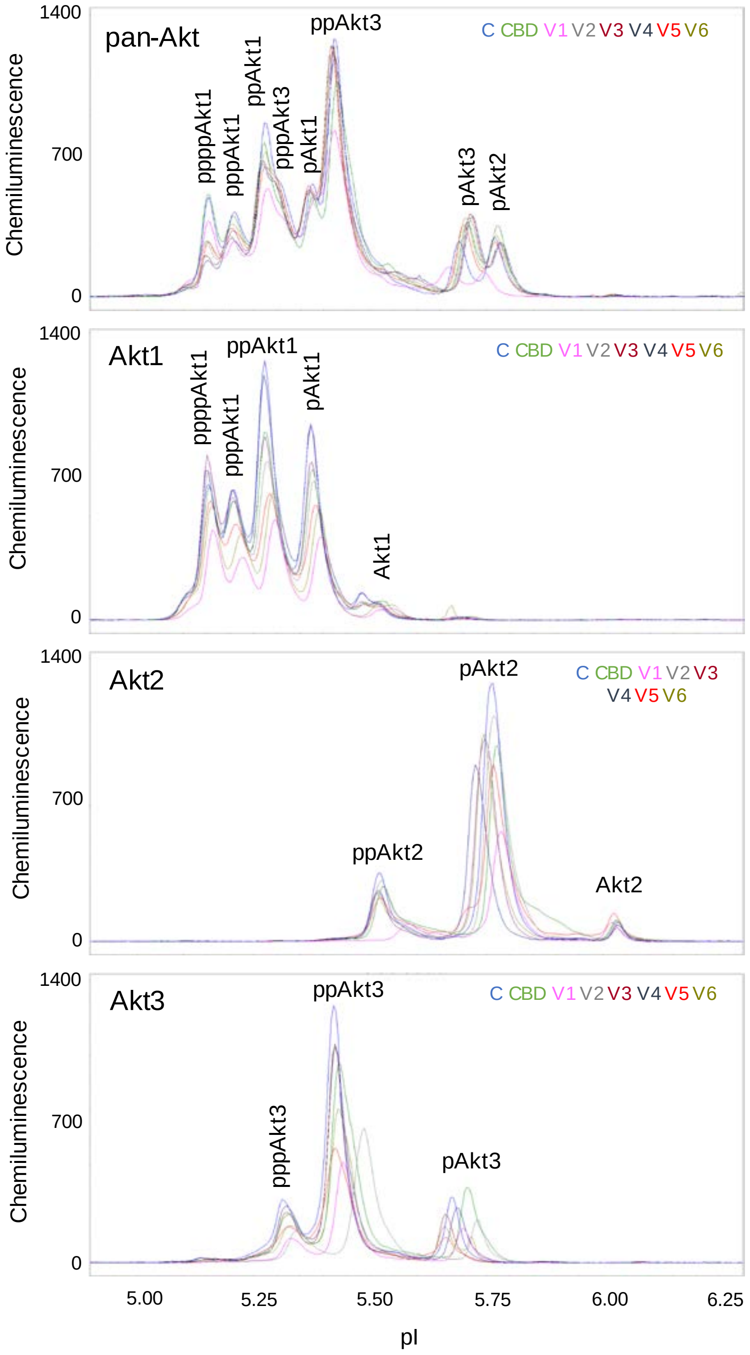

Supplemental Figure S1. Measuring the effects of isolated CBD and CBD oils on the expression and phosphorylation of Akt isoforms. Total Akt and Akt isoforms were identified in the SH-SY5Y cell lysates by cIEF immunoassays using antibodies against total Akt (pan-Akt, top panel), Akt1 (second panel), Akt2 (third panel), or Akt3 (fourth panel). The profiles of total Akt (pan-Akt), Akt1, Akt2, and Akt3 of SH-SY5Y cells before (C, blue) and after 24 hours of treatment with isolated CBD (6.25 µg/ml, green) or with CBD oil samples at 100 µg/ml final CBD concentration for V1 (pink), V2 (gray), V3 (deep red), V4 (dark blue), V5 (red), and V6 (dark yellow).

A

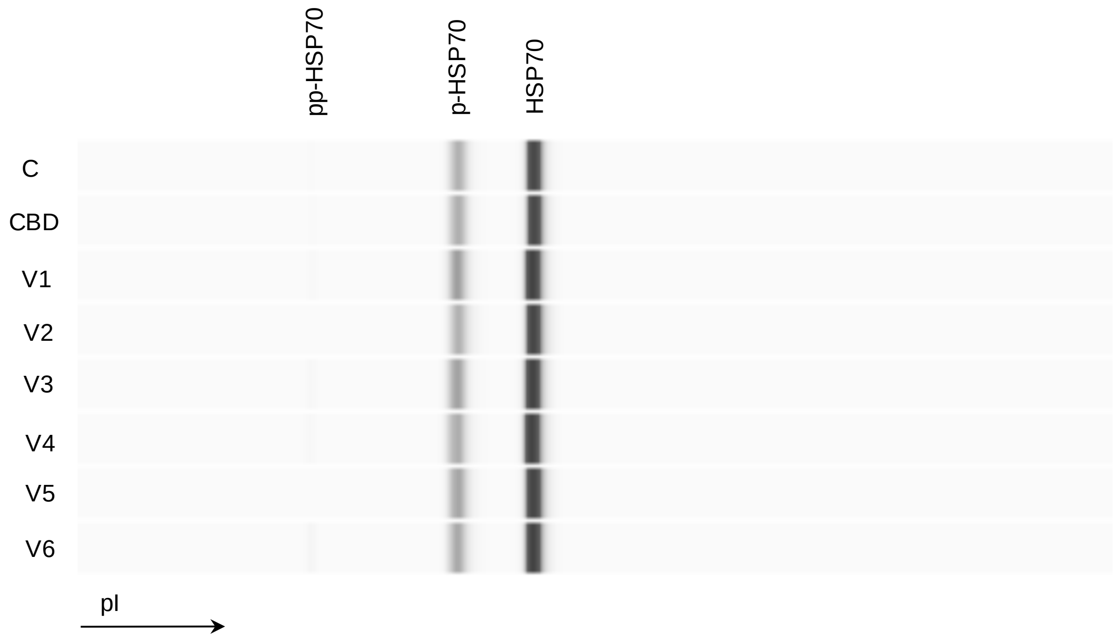

B

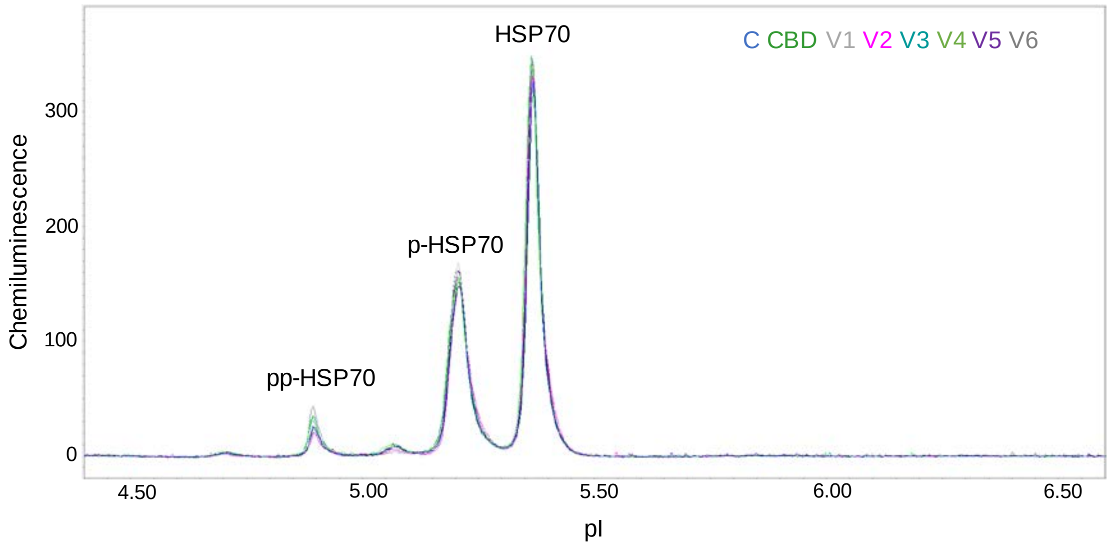

Supplemental Figure S2. HSP70 serves as a loading control for cIEF immunoassays. (**A**) Detection of HSP70 isoforms in individual capillaries. (**B**) Overlaid of quantitative graphical presentation of chemiluminescence data as a function of isoelectric points (pI). C, untreated control; CBD, isolated CBD; V1, CBD oil V1; V2, CBD oil V2; V3, CBD oil V3; V4, CBD oil V4; V5, CBD oil V5; V6, CBD oil V6.

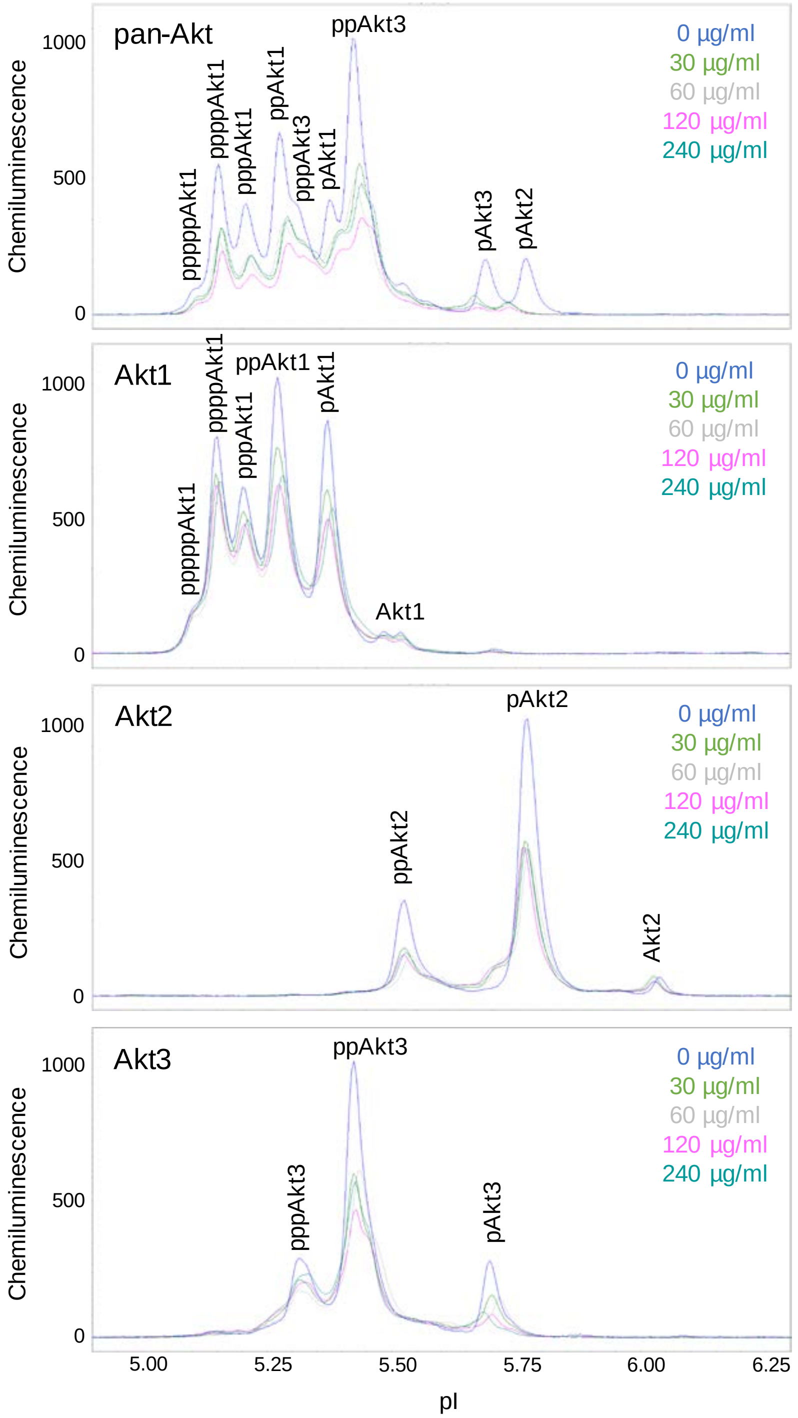

Supplemental Figure S3. Dose-dependent effects of CBD oil V1 on the expression and phosphorylation of Akt isoforms. Total Akt and Akt isoforms were identified in the SH-SY5Y cell lysates by cIEF immunoassays using antibodies against total Akt (pan-Akt, top panel), Akt1 (second panel), Akt2 (third panel), or Akt3 (fourth panel). The profiles of total Akt (pan-Akt), Akt1, Akt2, and Akt3 of SH-SY5Y cells before (0 µg/ml, blue) and after 24 hours of treatment with CBD oil V1 at final CBD concentrations of 30 µg/ml (green), 60 µg/ml (gray), 120 µg/ml (pink), and 240 µg/ml (dark teal).

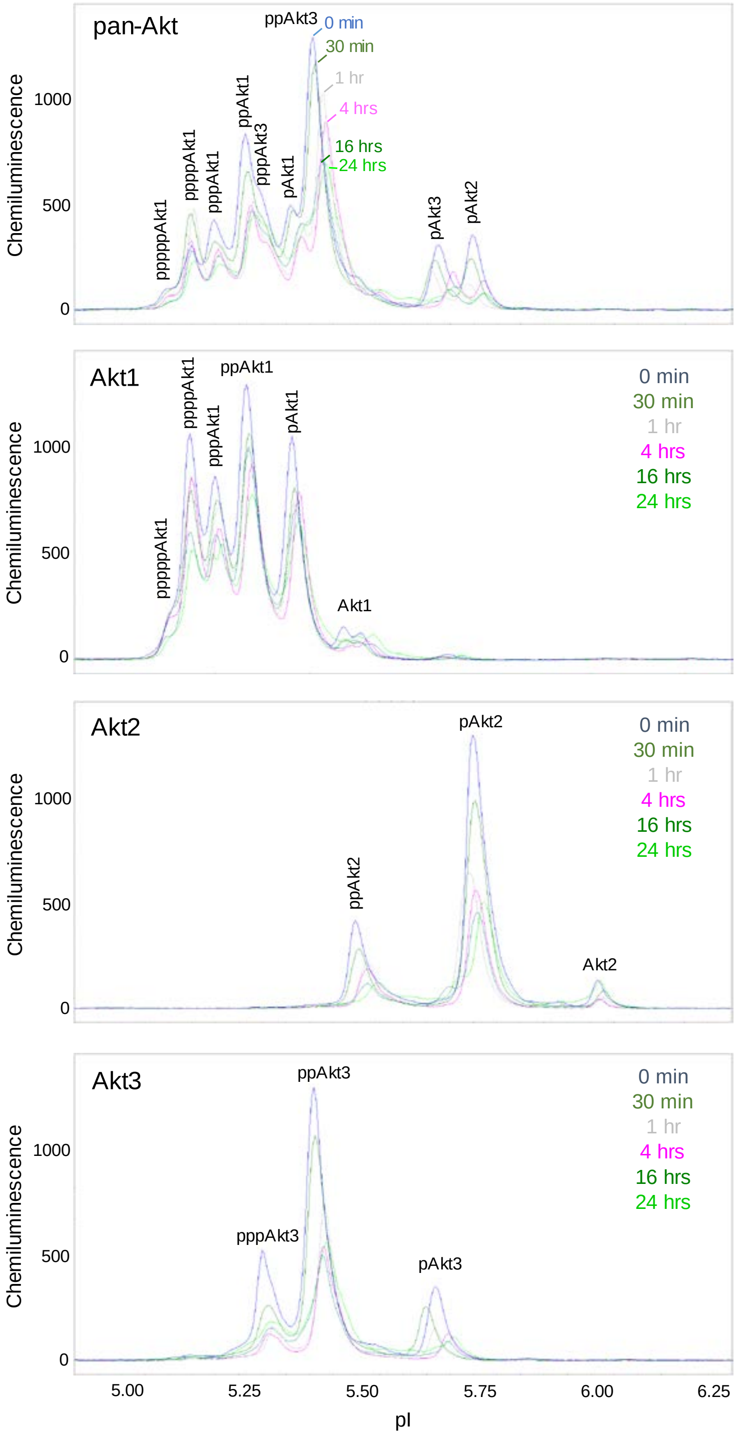

Supplemental Figure S4. Time-dependent effects of CBD oil V1 on the expression and phosphorylation of Akt isoforms. Total Akt and Akt isoforms were identified in SH-SY5Y cell lysates by cIEF immunoassays using antibodies against total Akt (pan-Akt, top panel), Akt1 (second panel), Akt2 (third panel), or Akt3 (fourth panel). The profiles of total Akt (pan-Akt), Akt1, Akt2, and Akt3 of SH-SY5Y cells before (0 min, blue) and after treatment with CBD oil V1 at a final CBD concentration of 100 µg/ml at 30 min (green), 1 hr (grey), 4 hrs (pink), 16 hrs (dark green), and 24 hrs (light green).

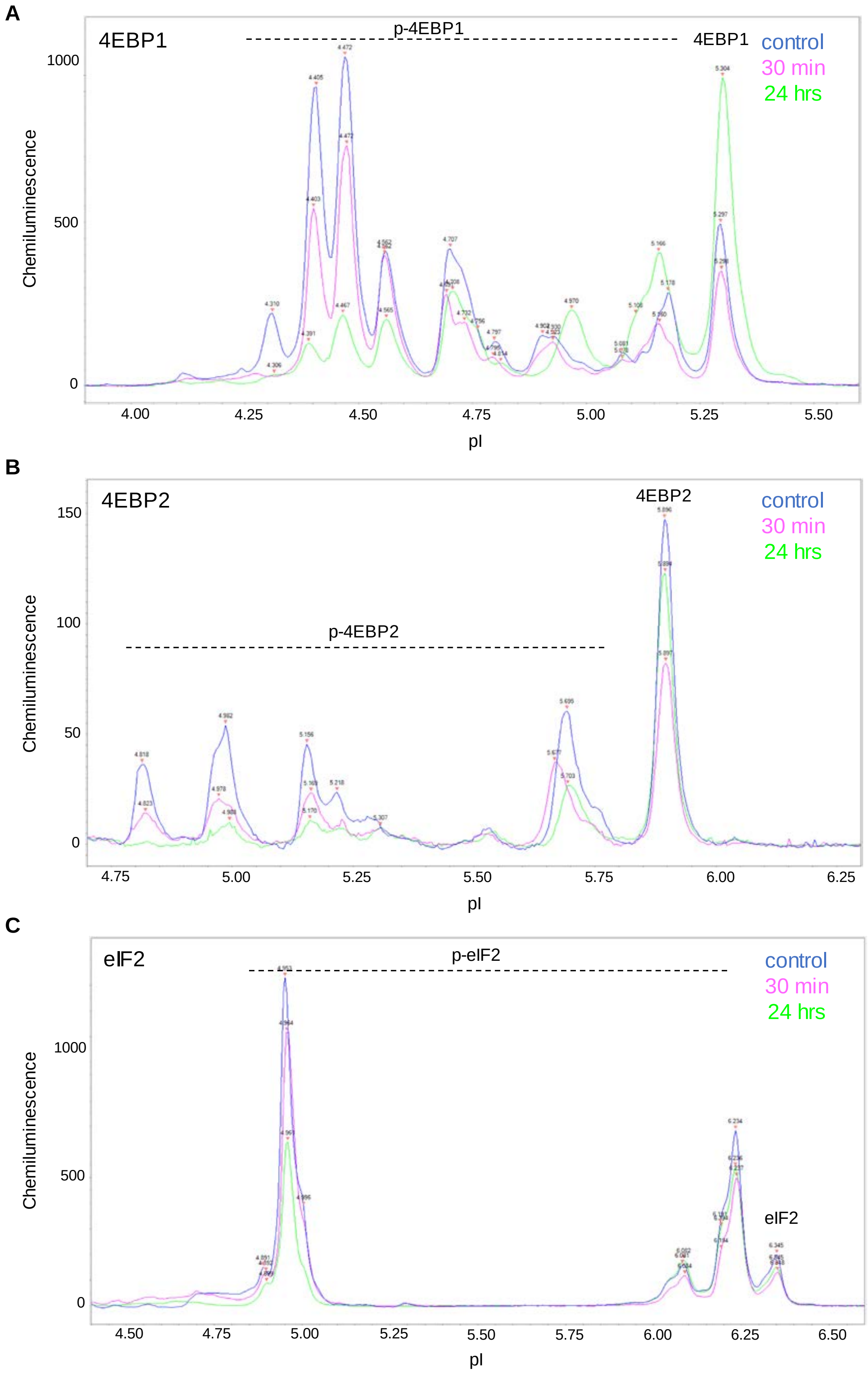
A
4EBP1
p-4EBP1
4EBP1
control
30 min
24 hrs
Chemiluminescence
1000
500
0
4.00
4.25
4.50
4.75
5.00
5.25
5.50
pI
B
4EBP2
4EBP2
control
30 min
24 hrs
p-4EBP2
Chemiluminescence
150
100
50
0
4.75
5.00
5.25
5.50
5.75
6.00
6.25
pI
C
eIF2
p-eIF2
control
30 min
24 hrs
eIF2
Chemiluminescence
1000
500
0
4.50
4.75
5.00
5.25
5.50
5.75
6.00
6.25
6.50
pI

D

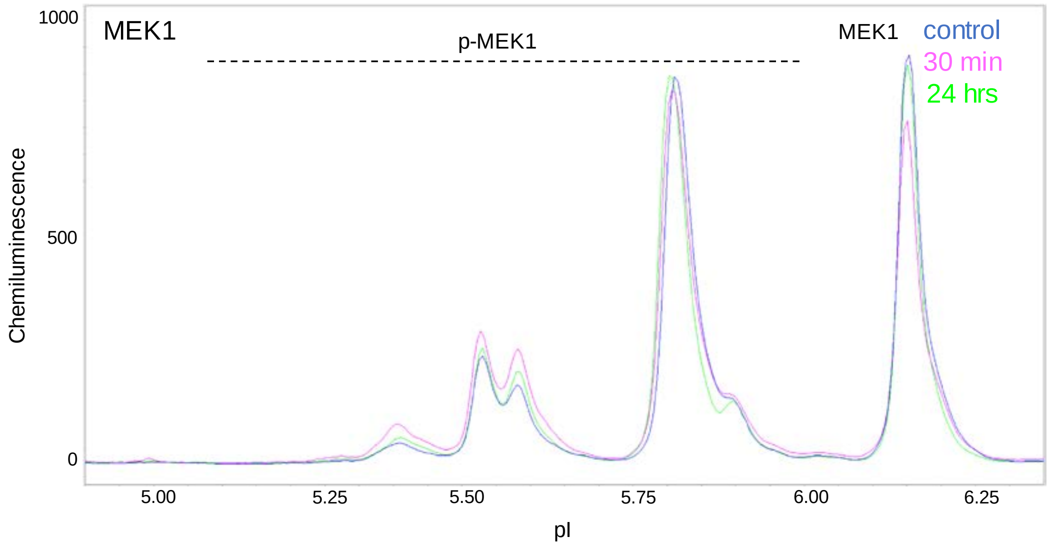
MEK1
p-MEK1
MEK1
control
30 min
24 hrs
1000
500
0
Chemiluminescence
5.00
5.25
5.50
5.75
6.00
6.25
pI

E

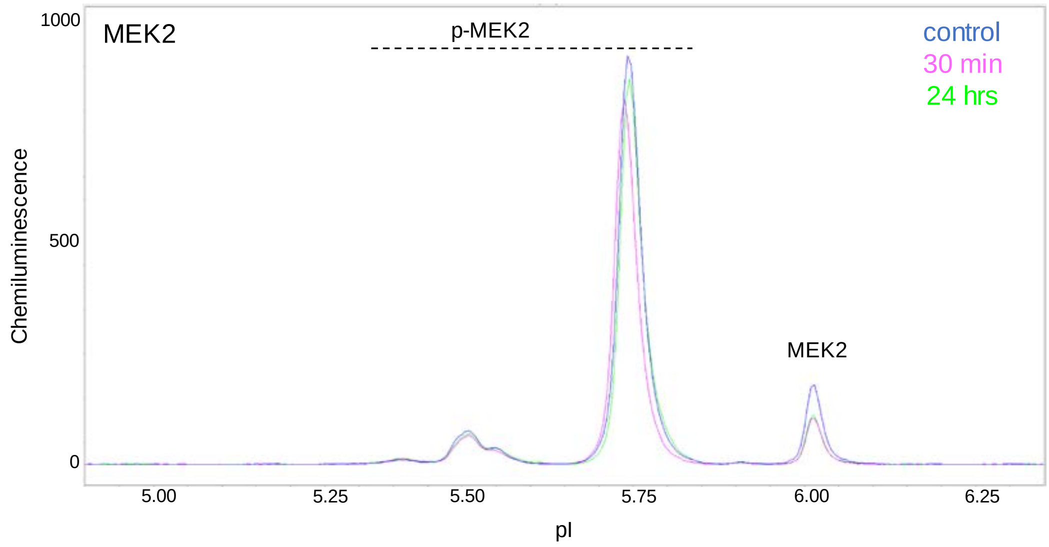
MEK2
p-MEK2
MEK2
control
30 min
24 hrs
1000
500
0
Chemiluminescence
5.00
5.25
5.50
5.75
6.00
6.25
pI

F

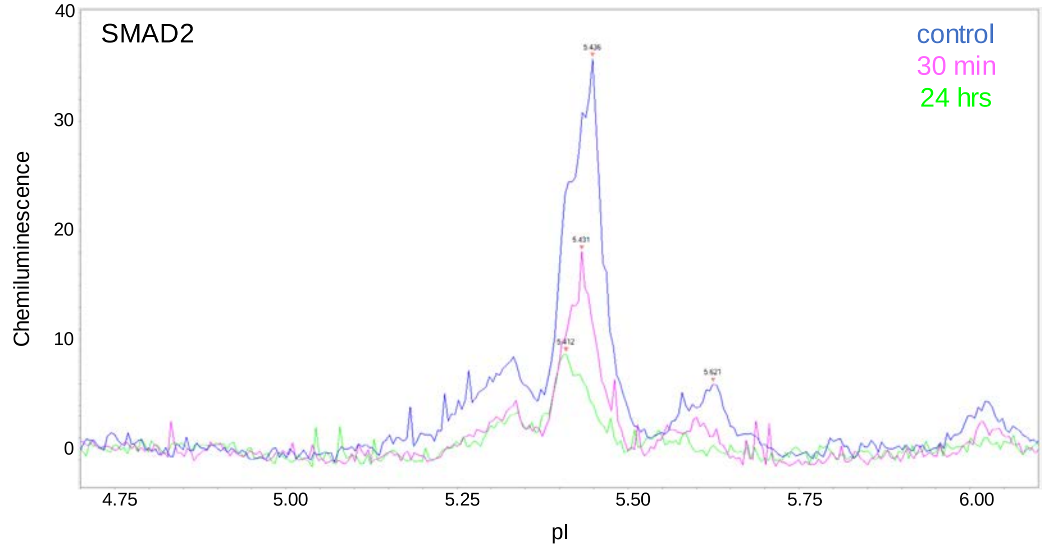
SMAD2
control
30 min
24 hrs
5.436
5.431
5.412
5.621
40
30
20
10
0
Chemiluminescence
4.75
5.00
5.25
5.50
5.75
6.00
pI

G

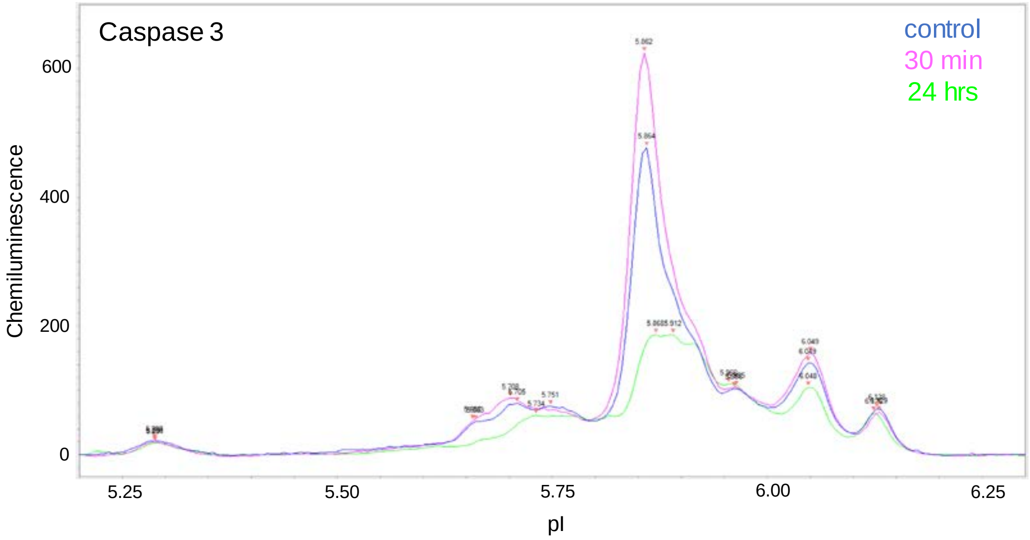
Caspase 3
control
30 min
24 hrs
Chemiluminescence
600
400
200
0
5.25
5.50
5.75
6.00
6.25
pI

H

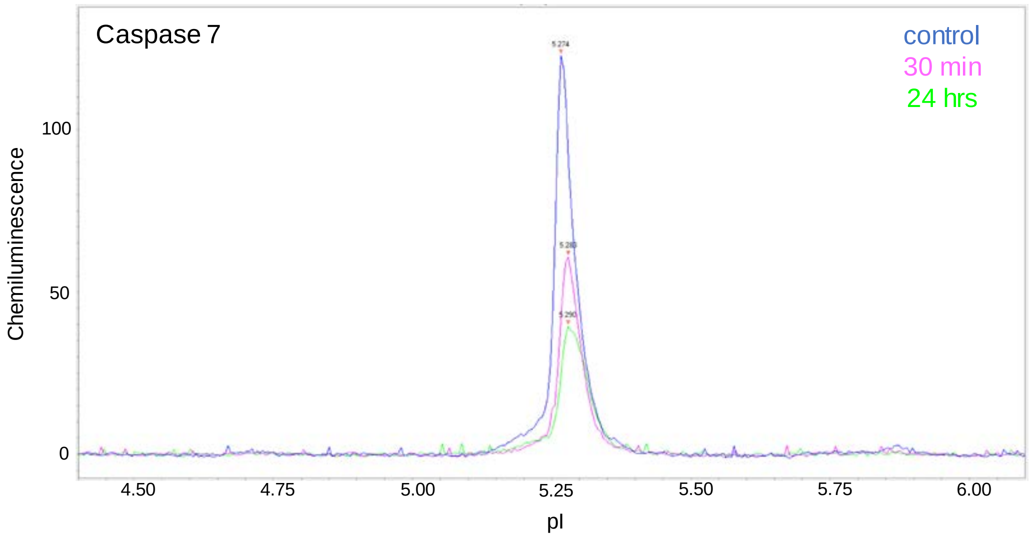
Caspase 7
control
30 min
24 hrs
Chemiluminescence
100
50
0
4.50
4.75
5.00
5.25
5.50
5.75
6.00
pI

I

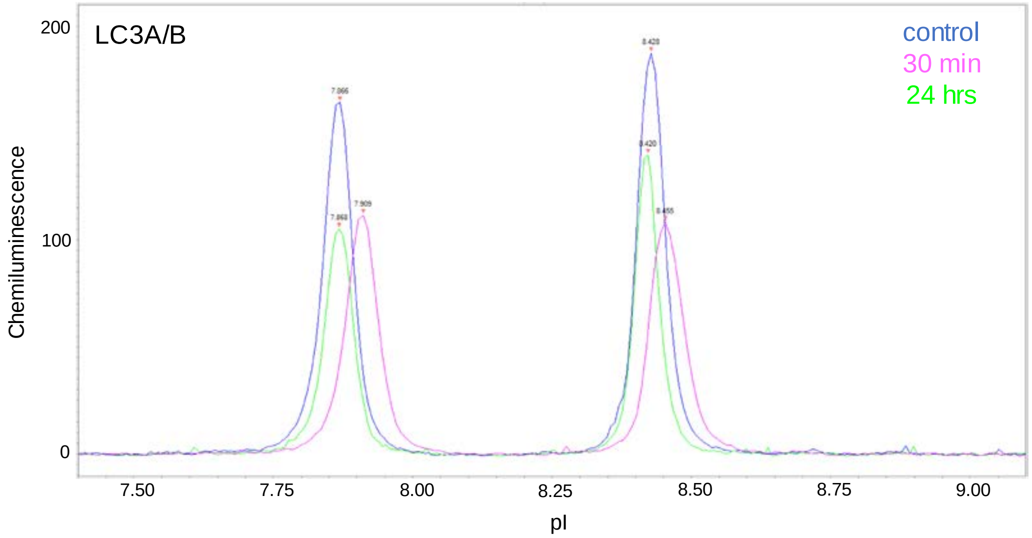
LC3A/B
control
30 min
24 hrs
Chemiluminescence
200
100
0
7.50
7.75
8.00
8.25
8.50
8.75
9.00
pI

J

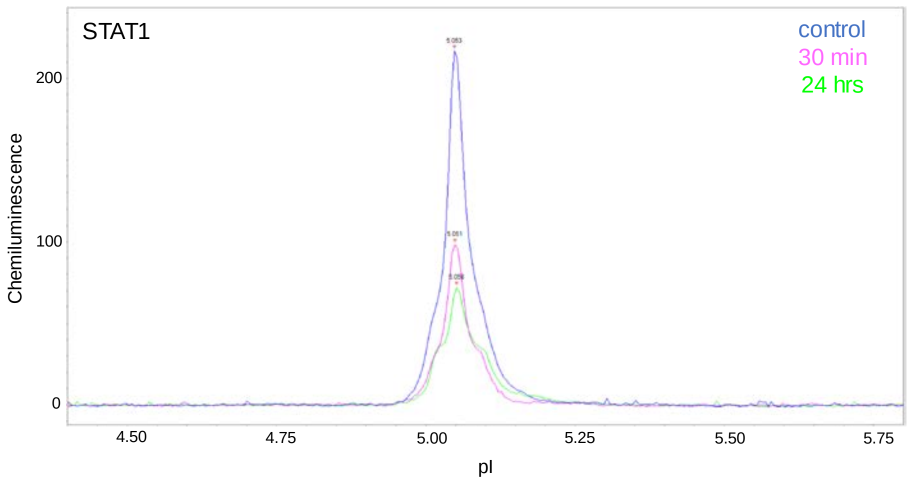

K

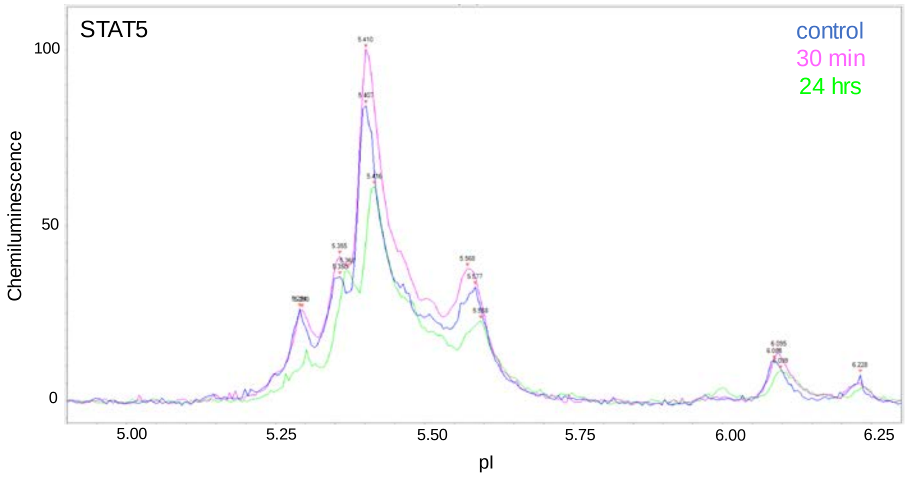

L

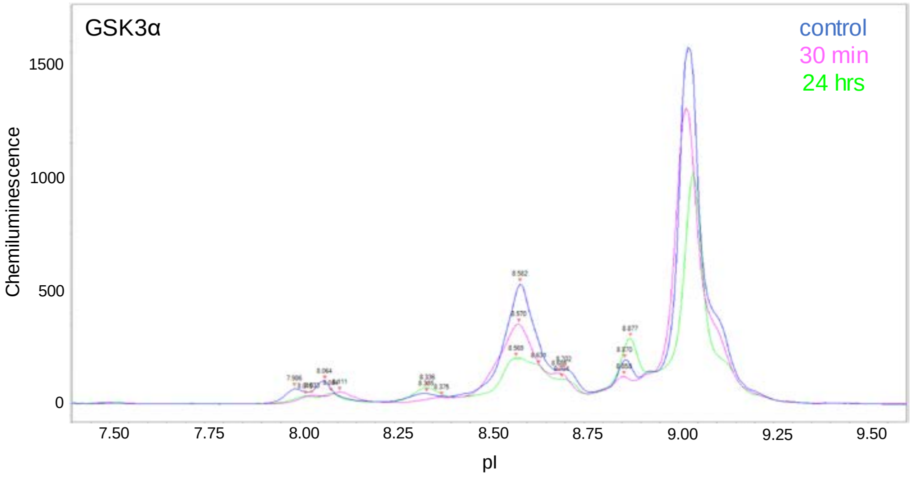

Supplemental Figure S5. Proteomic profiling of the effects of CBD oil V1 with multiplexed cIEF immunoassays. Selective profiles of SH-SY5Y proteins following the treatment with 100 µg/ml of final CBD concentration at time 0 minute (blue), 30 minutes (pink), and 24 hours (green) for: (**A**) 4EBP1, (**B**) 4EBP2, (**C**) eIF2, (**D**) MEK1, (**E**) MEK2, (**F**) SMAD2, (**G**) Caspase 3, (**H**) Caspase 7, (**I**) LC3A/B, (**J**) STAT1, (**K**) STAT5, and (**L**) GSK3α.

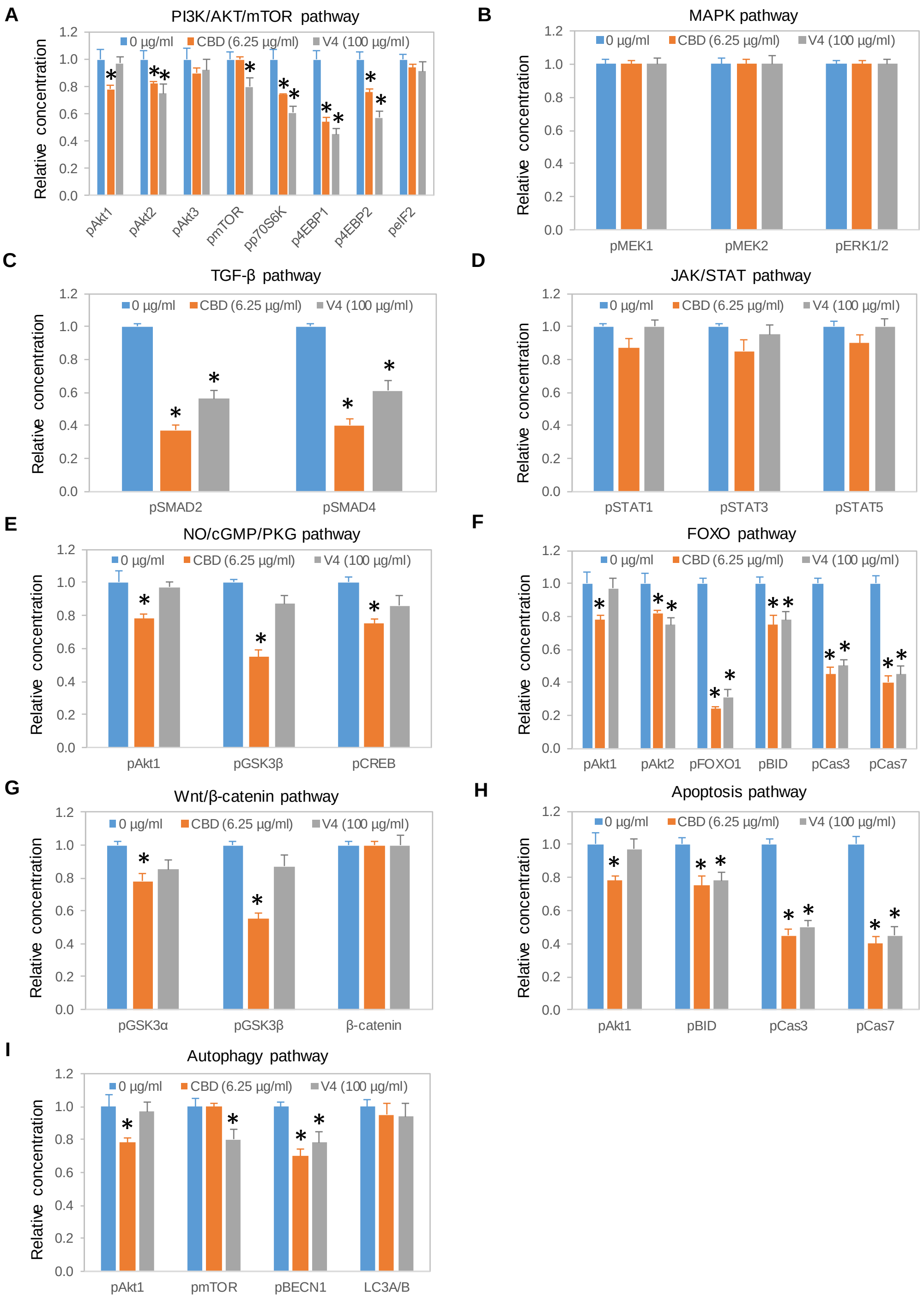

Supplemental Figure S6. Expression of protein isoforms in selected signaling pathways following treatment with isolated CBD and CBD oil V4. (**A**) PI3K/Akt/mTOR pathway, (**B**) MAPK pathway, (**C**) TGF-β pathway, (**D**) JAK/STAT pathway, (**E**) NO/cGMP/PKG pathway, (**F**) FOXO pathway, (**G**) Wnt/β-catenin pathway, (**H**) apoptosis pathway, and (**I**) autophagy pathway. Blue, orange, and grey colors are untreated controls (0 µg/ml) and treated with CBD isolate (6.25 µg/ml) and treated with CBD oil V4 (100 µg/ml of final CBD concentration) for 24 hours, respectively. Error bars are standard deviation across six repeated measurements per experimental condition. Asterisks indicate statistical significance for $p \leq 0.05$ versus control.

Supplemental Table S1. List of primary and secondary antibodies

№	Antibody	Cat. No.	Vendor
1	pan-Akt	8312	Santa Cruz Biotech (Dallas, TX)
2	Akt1	2938	Cell Signaling (Danvers, MA)
3	Akt2	3063	Cell Signaling
4	Akt3	8018	Cell Signaling
5	mTOR	2983	Cell Signaling
6	pmTOR (Ser2448)	5536	Cell Signaling
7	p70S6K	9202	Cell Signaling
8	pp70S6K (Thr389)	9234	Cell Signaling
9	4EBP1	9644	Cell Signaling
10	4EBP2	2845	Cell Signaling
11	eIF2	9722	Cell Signaling
12	MEK1	07-641	Millipore (Billerica, MA)
13	MEK2	9125	Cell Signaling
14	ERK1/2	040-474	Protein Simple (Santa Clara, CA)
15	STAT1	14994	Cell Signaling
16	STAT3	4904	Cell Signaling
17	STAT5	94205	Cell Signaling
18	FOXO1	2880	Cell Signaling
19	pFOXO1 (Thr24)	9464	Cell Signaling
20	BID	2002	Cell Signaling
21	Caspase 3	9665	Cell Signaling
22	Caspase 7	12827	Cell Signaling
23	CREB	9104	Cell Signaling
24	pCREB (Ser133)	9198	Cell Signaling
25	SMAD2	5339	Cell Signaling
26	SMAD4	38454	Cell Signaling
27	Beclin 1	NB500-249	Novus (Centennial, CO)
28	LC3A/B	12741	Cell Signaling
29	β-catenin	NBP1-54467	Novus
30	GSK3α	4337	Cell Signaling
31	GSK3β	9315	Cell Signaling
32	β-actin	MAB8929	R&D Systems (Minneapolis, MN)
33	HSP60	F1800	R&D Systems
34	HSP70	4872	Cell Signaling
35	Secondary antibody (anti-rabbit HRP)	040-656	Protein Simple
36	Secondary antibody (anti-rabbit HRP)	042-206	Protein Simple
37	Secondary antibody (anti-mouse HRP)	042-205	Protein Simple
38	Secondary antibody (anti-rabbit NIR)	043-819	Protein Simple
39	Secondary antibody (anti-mouse NIR)	043-821	Protein Simple

Supplemental Table S2. List of biomarker proteins and their functions

№	Protein	Name	Function
1	Akt1	Protein kinase B, isoform 1	Apoptosis, proliferation, & cell migration
2	Akt2	Protein kinase B, isoform 2	Glucose metabolism
3	Akt3	Protein kinase B, isoform 3	Neuronal development
4	mTOR	Mechanistic target of rapamycin	Proliferation, motility, survival, autophagy,
5	p70S6K	Ribosomal S6 kinase β-1	Protein synthesis
6	4EBP1	Eukaryotic translation initiation factor 4E-binding protein 1	Protein synthesis
7	4EBP2	Eukaryotic translation initiation factor 4E-binding protein 2	Protein synthesis
8	eIF2	Eukaryotic initiation factor 2	Protein synthesis
9	MEK1	Mitogen-activated protein kinase kinase 1	Proliferation, differentiation, development
10	MEK2	Mitogen-activated protein kinase kinase 2	Proliferation, differentiation, development
11	ERK1/2	Mitogen-activated protein kinase 1/2	Proliferation, differentiation, development
12	STAT1	Signal transducer and activator of transcription 1	Immunity, proliferation, differentiation
13	STAT3	Signal transducer and activator of transcription 3	Immunity, proliferation, differentiation
14	STAT5	Signal transducer and activator of transcription 5	Immunity, proliferation, differentiation
15	FOXO1	Forkhead box protein O, transcription factor	Gluconeogenesis, glycogenolysis, apoptosis
16	BID	BH3 interacting-domain death	Pro-apoptotic member of Bcl-2 family
17	Caspase 3	Cysteine-aspartic acid protease	Apoptosis
18	Caspase 7	Cysteine-aspartic acid protease	Apoptosis
19	SMAD2	Signal transducer for the receptors of TGF-β family	Growth and development
20	SMAD4	Signal transducer for the receptors of TGF-β family	Growth and development
21	CREB	cAMP response element-binding protein	Long-term memory formation, neuronal survival
22	GSK3α	Glycogen synthase kinase 3 α	Energy metabolism, neuronal cell development, body pattern formation
23	GSK3β	Glycogen synthase kinase 3 β	Energy metabolism, neuronal cell development, body pattern formation
24	β-catenin	β-catenin	Cell-cell adhesion, transcription, synaptic plasticity
25	Beclin 1	ATG6 autophagy related 6 homolog	Tumorigenesis, neurodegeneration, autophagic programmed cell death
26	LC3A/B	Microtubule-associated protein 1A/B light chain 3B	Autophagosome biogenesis

Supplemental data. The following pages contain the cannabinoid and terpene profiles of six CBD oils V1-V6, which were analyzed by three independent laboratories at Botanacor, Aromatic Plant Research Center, and dōTERRA.

botanacor

CERTIFICATE OF ANALYSIS

prepared for: ROSEMAN UNIVERSITY
10530 DISCOVERY DRIVE
LAS VEGAS, NV 89135

V1

**Batch ID:**	OHO-CO2	**Test ID:**	7708631.0051
**Reported:**	24-Sep-2019	**Method:**	TM14
**Type:**	Concentrate		
**Test:**	Potency		

CANNABINOID PROFILE

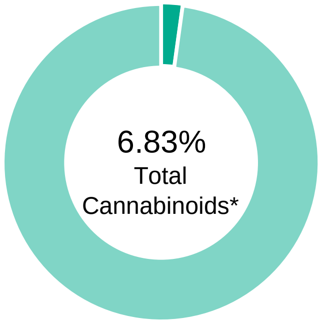

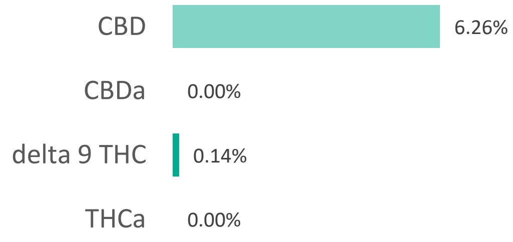

% = % (w/w) = Percent (Weight of Analyte / Weight of Product)

* Total Cannabinoids result reflects the absolute sum of all cannabinoids detected.

** Total Potential THC/CBD is calculated using the following formulas to take into account the loss of a carboxyl group during decarboxylation step.

Total THC = THC + (THCa *(0.877)) and Total CBD = CBD + (CBDa *(0.877))

Compound	LOQ (%)	Result (%)	Result (mg/g)
Delta 9-Tetrahydrocannabinolic acid (THCA-A)	0.07	0.00	0.0
Delta 9-Tetrahydrocannabinol (Delta 9THC)	0.04	0.14	1.4
Cannabidiolic acid (CBDA)	0.07	0.00	0.0
Cannabidiol (CBD)	0.04	6.26	62.6
Delta 8-Tetrahydrocannabinol (Delta 8THC)	0.04	0.00	0.0
Cannabinolic Acid (CBNA)	0.10	0.00	0.0
Cannabinol (CBN)	0.04	0.00	0.0
Cannabigerolic acid (CBGA)	0.06	0.00	0.0
Cannabigerol (CBG)	0.04	0.15	1.5
Tetrahydrocannabivarinic Acid (THCVA)	0.06	0.00	0.0
Tetrahydrocannabivarin (THCV)	0.03	0.00	0.0
Cannabidivarinic Acid (CBDVA)	0.07	0.00	0.0
Cannabidivarin (CBDV)	0.04	0.06	0.6
Cannabichromenic Acid (CBCA)	0.05	0.00	0.0
Cannabichromene (CBC)	0.07	0.22	2.2
**Total Cannabinoids**		**6.83**	**68.30**
Total Potential THC**		0.14	1.40
Total Potential CBD**		6.26	62.60

NOTES:
N/A

FINAL APPROVAL

PREPARED BY / DATE	APPROVED BY / DATE
Daniel Weidensaul<br>24-Sep-2019<br>3:25 PM	David Green<br>24-Sep-2019<br>4:17 PM

Testing results are based solely upon the sample submitted to Botanacor Laboratories, LLC, in the condition it was received. Botanacor Laboratories, LLC warrants that all analytical work is conducted professionally in accordance with all applicable standard laboratory practices using validated methods. Data was generated using an unbroken chain of comparison to NIST traceable Reference Standards and Certified Reference Materials. This report may not be reproduced, except in full, without the written approval of Botanacor Laboratories, LLC. ISO/IEC 17025:2005 Accredited A2LA Certificate Number 4329.02

Certificate #4329.02

CERTIFICATE OF ANALYSIS

prepared for: ROSEMAN UNIVERSITY
10530 DISCOVERY DRIVE
LAS VEGAS, NV 89135

V1

**Batch ID:**	OHO-CO2	**Test ID:**	9019026.0020
**Reported:**	20-Sep-2019	**Method:**	TM10
**Type:**	Concentrate		
**Test:**	Terpenes		

TERPENE PROFILE

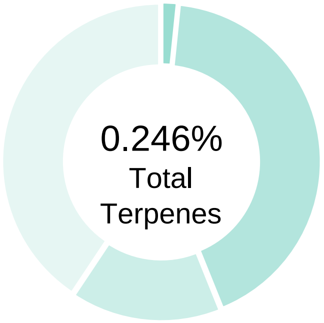

Compound	%(w/w)	mg/g
(-)-alpha-Bisabolol	0.100	1
Camphene	0.000	0
delta-3-Carene	0.000	0
beta-Caryophyllene	0.104	1.04
(-)-Caryophyllene Oxide	0.000	0
p-Cymene	0.000	0
Eucalyptol	0.000	0
Geraniol	0.000	0
alpha-Humulene	0.038	0.38
(-)-Isopulegol	0.000	0
d-Limonene	0.000	0
Linalool	0.004	0.04
beta-Myrcene	0.000	0
cis-Nerolidol	0.000	0
trans-Nerolidol	0.000	0
Ocimene	0.000	0
beta-Ocimene	0.000	0
alpha-Pinene	0.000	0
(-)-beta-Pinene	0.000	0
alpha-Terpinene	0.000	0
gamma-Terpinene	0.000	0
Terpinolene	0.000	0
	**0.246%**	**2.46**

PREDOMINANT TERPENES

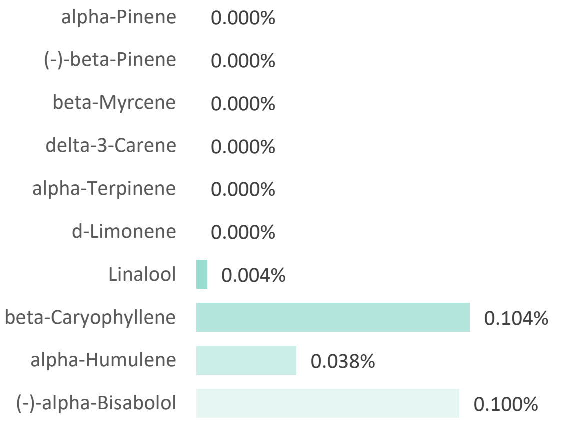

NOTES:
0

FINAL APPROVAL

Daniel Weidensaul<br>20-Sep-2019<br>10:53 AM	David Green<br>20-Sep-2019<br>11:17 AM
PREPARED BY / DATE	APPROVED BY / DATE

Testing results are based solely upon the sample submitted to Botanacor Laboratories, LLC. Botanacor Laboratories, LLC warrants that all analytical work is conducted professionally in accordance with all applicable standard laboratory practices using validated methods. Data was generated using an unbroken chain of comparison to NIST traceable Reference Standards and Certified Reference Materials. This report may not be reproduced, except in full, without the written approval of Botanacor Laboratories, LLC. ISO/IEC 17025:2005 Accredited A2LA Certificate Number 4329.02

botanacor

CERTIFICATE OF ANALYSIS

prepared for: ROSEMAN UNIVERSITY
10530 DISCOVERY DRIVE
LAS VEGAS, NV 89135

V2

**Batch ID:**	OVHSO-CO2	**Test ID:**	7708631.0053
**Reported:**	24-Sep-2019	**Method:**	TM14
**Type:**	Concentrate		
**Test:**	Potency		

CANNABINOID PROFILE

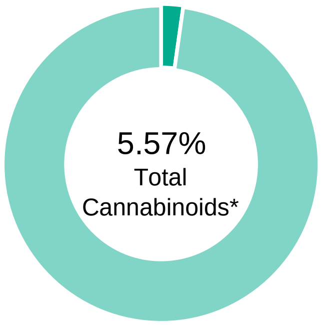

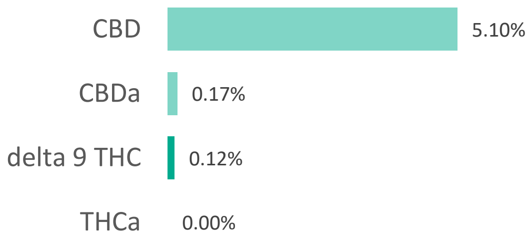

Compound	LOQ (%)	Result (%)	Result (mg/g)
Delta 9-Tetrahydrocannabinolic acid (THCA-A)	0.07	0.00	0.0
Delta 9-Tetrahydrocannabinol (Delta 9THC)	0.04	0.12	1.2
Cannabidiolic acid (CBDA)	0.07	0.17	1.7
Cannabidiol (CBD)	0.04	5.10	51.0
Delta 8-Tetrahydrocannabinol (Delta 8THC)	0.04	0.00	0.0
Cannabinolic Acid (CBNA)	0.10	0.00	0.0
Cannabinol (CBN)	0.04	0.00	0.0
Cannabigerolic acid (CBGA)	0.06	0.00	0.0
Cannabigerol (CBG)	0.04	0.00	0.0
Tetrahydrocannabivarinic Acid (THCVA)	0.06	0.00	0.0
Tetrahydrocannabivarin (THCV)	0.03	0.00	0.0
Cannabidivarinic Acid (CBDVA)	0.07	0.00	0.0
Cannabidivarin (CBDV)	0.04	0.00	0.0
Cannabichromenic Acid (CBCA)	0.06	0.00	0.0
Cannabichromene (CBC)	0.07	0.18	1.8
**Total Cannabinoids**		**5.57**	**55.70**
Total Potential THC**		0.12	1.20
Total Potential CBD**		5.25	52.49

% = % (w/w) = Percent (Weight of Analyte / Weight of Product)

* Total Cannabinoids result reflects the absolute sum of all cannabinoids detected.

** Total Potential THC/CBD is calculated using the following formulas to take into account the loss of a carboxyl group during decarboxylation step.

Total THC = THC + (THCa *(0.877)) and Total CBD = CBD + (CBDa *(0.877))

NOTES:
N/A

FINAL APPROVAL

Daniel Weidensaul<br>24-Sep-2019<br>3:25 PM	David Green<br>24-Sep-2019<br>4:17 PM
PREPARED BY / DATE	APPROVED BY / DATE

Testing results are based solely upon the sample submitted to Botanacor Laboratories, LLC, in the condition it was received. Botanacor Laboratories, LLC warrants that all analytical work is conducted professionally in accordance with all applicable standard laboratory practices using validated methods. Data was generated using an unbroken chain of comparison to NIST traceable Reference Standards and Certified Reference Materials. This report may not be reproduced, except in full, without the written approval of Botanacor Laboratories, LLC. ISO/IEC 17025:2005 Accredited A2LA Certificate Number 4329.02

ilac-MRA ACCREDITED
Certificate #4329.02

botanacor

CERTIFICATE OF ANALYSIS

prepared for: ROSEMAN UNIVERSITY
10530 DISCOVERY DRIVE
LAS VEGAS, NV 89135

V2

**Batch ID:**	OVHSO-CO2	**Test ID:**	9019026.0022
**Reported:**	20-Sep-2019	**Method:**	TM10
**Type:**	Concentrate		
**Test:**	Terpenes		

TERPENE PROFILE

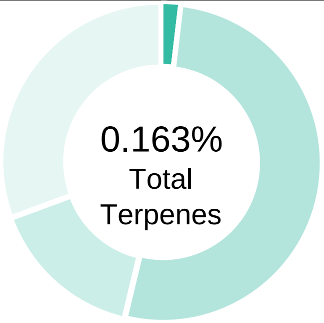

Compound	%(w/w)	mg/g
(-)-alpha-Bisabolol	0.049	0.49
Camphene	0.000	0
delta-3-Carene	0.000	0
beta-Caryophyllene	0.083	0.83
(-)-Caryophyllene Oxide	0.000	0
p-Cymene	0.000	0
Eucalyptol	0.003	0.03
Geraniol	0.000	0
alpha-Humulene	0.025	0.25
(-)-Isopulegol	0.000	0
d-Limonene	0.000	0
Linalool	0.000	0
beta-Myrcene	0.003	0.03
cis-Nerolidol	0.000	0
trans-Nerolidol	0.000	0
Ocimene	0.000	0
beta-Ocimene	0.000	0
alpha-Pinene	0.000	0
(-)-beta-Pinene	0.000	0
alpha-Terpinene	0.000	0
gamma-Terpinene	0.000	0
Terpinolene	0.000	0
	**0.163%**	**1.63**

PREDOMINANT TERPENES

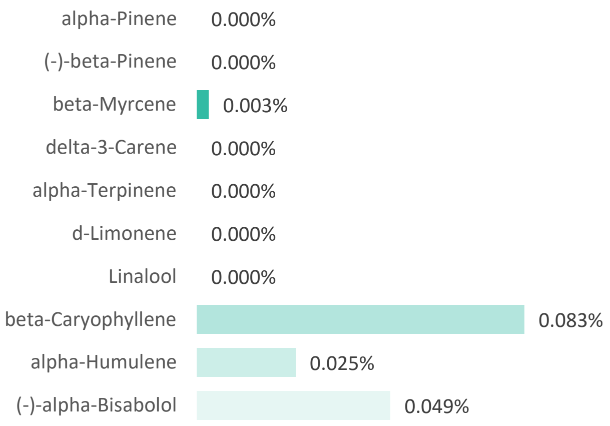

NOTES:
0

FINAL APPROVAL

Daniel Weidensaul<br>20-Sep-2019<br>10:53 AM	David Green<br>20-Sep-2019<br>11:17 AM
PREPARED BY / DATE	APPROVED BY / DATE

Testing results are based solely upon the sample submitted to Botanacor Laboratories, LLC. Botanacor Laboratories, LLC warrants that all analytical work is conducted professionally in accordance with all applicable standard laboratory practices using validated methods. Data was generated using an unbroken chain of comparison to NIST traceable Reference Standards and Certified Reference Materials. This report may not be reproduced, except in full, without the written approval of Botanacor Laboratories, LLC. ISO/IEC 17025:2005 Accredited A2LA Certificate Number 4329.02

botanacor

CERTIFICATE OF ANALYSIS

prepared for: ROSEMAN UNIVERSITY

10530 DISCOVERY DRIVE
LAS VEGAS, NV 89135

V3

**Batch ID:**	MCY-CO2	**Test ID:**	7708631.0055
**Reported:**	24-Sep-2019	**Method:**	TM14
**Type:**	Concentrate		
**Test:**	Potency		

CANNABINOID PROFILE

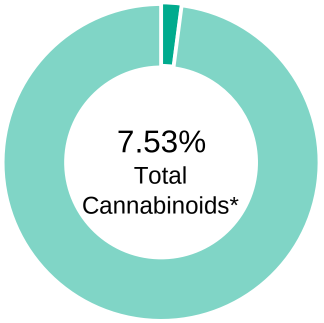

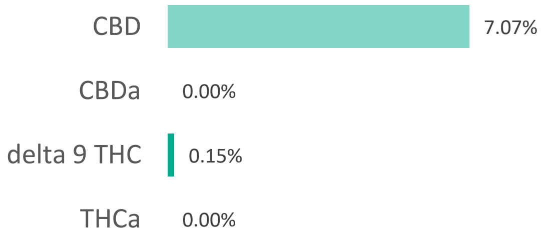

Compound	LOQ (%)	Result (%)	Result (mg/g)
Delta 9-Tetrahydrocannabinolic acid (THCA-A)	0.07	0.00	0.0
Delta 9-Tetrahydrocannabinol (Delta 9THC)	0.04	0.15	1.5
Cannabidiolic acid (CBDA)	0.07	0.00	0.0
Cannabidiol (CBD)	0.04	7.07	70.7
Delta 8-Tetrahydrocannabinol (Delta 8THC)	0.04	0.00	0.0
Cannabinolic Acid (CBNA)	0.10	0.00	0.0
Cannabinol (CBN)	0.04	0.00	0.0
Cannabigerolic acid (CBGA)	0.06	0.00	0.0
Cannabigerol (CBG)	0.04	0.12	1.2
Tetrahydrocannabivarinic Acid (THCVA)	0.06	0.00	0.0
Tetrahydrocannabivarin (THCV)	0.03	0.00	0.0
Cannabidivarinic Acid (CBDVA)	0.07	0.00	0.0
Cannabidivarin (CBDV)	0.04	0.00	0.0
Cannabichromenic Acid (CBCA)	0.05	0.00	0.0
Cannabichromene (CBC)	0.06	0.19	1.9
**Total Cannabinoids**		**7.53**	**75.30**
Total Potential THC**		0.15	1.50
Total Potential CBD**		7.07	70.70

% = % (w/w) = Percent (Weight of Analyte / Weight of Product)

* Total Cannabinoids result reflects the absolute sum of all cannabinoids detected.

** Total Potential THC/CBD is calculated using the following formulas to take into account the loss of a carboxyl group during decarboxylation step.

Total THC = THC + (THCa *(0.877)) and Total CBD = CBD + (CBDa *(0.877))

NOTES:

N/A

FINAL APPROVAL

PREPARED BY / DATE	APPROVED BY / DATE
Daniel Weidensaul<br>24-Sep-2019<br>3:25 PM	David Green<br>24-Sep-2019<br>4:17 PM

Testing results are based solely upon the sample submitted to Botanacor Laboratories, LLC, in the condition it was received. Botanacor Laboratories, LLC warrants that all analytical work is conducted professionally in accordance with all applicable standard laboratory practices using validated methods. Data was generated using an unbroken chain of comparison to NIST traceable Reference Standards and Certified Reference Materials. This report may not be reproduced, except in full, without the written approval of Botanacor Laboratories, LLC. ISO/IEC 17025:2005 Accredited A2LA Certificate Number 4329.02

ilac-MRA A2LA ACCREDITED

Certificate #4329.02

botanacor

CERTIFICATE OF ANALYSIS

prepared for: ROSEMAN UNIVERSITY

10530 DISCOVERY DRIVE
LAS VEGAS, NV 89135

V3

**Batch ID:**	MCY-CO2	**Test ID:**	9019026.0024
**Reported:**	20-Sep-2019	**Method:**	TM10
**Type:**	Concentrate		
**Test:**	Terpenes		

TERPENE PROFILE

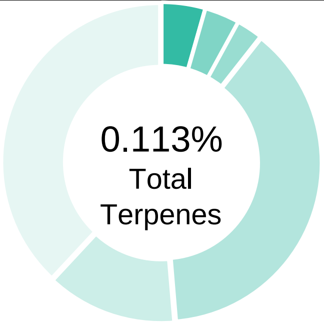

PREDOMINANT TERPENES

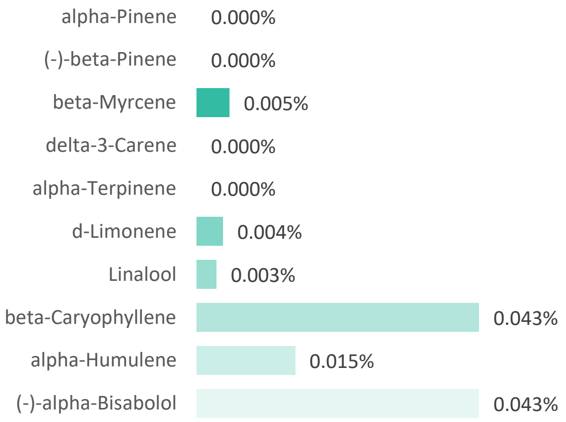

Compound	%(w/w)	mg/g
(-)-alpha-Bisabolol	0.043	0.43
Camphene	0.000	0
delta-3-Carene	0.000	0
beta-Caryophyllene	0.043	0.43
(-)-Caryophyllene Oxide	0.000	0
p-Cymene	0.000	0
Eucalyptol	0.000	0
Geraniol	0.000	0
alpha-Humulene	0.015	0.15
(-)-Isopulegol	0.000	0
d-Limonene	0.004	0.04
Linalool	0.003	0.03
beta-Myrcene	0.005	0.05
cis-Nerolidol	0.000	0
trans-Nerolidol	0.000	0
Ocimene	0.000	0
beta-Ocimene	0.000	0
alpha-Pinene	0.000	0
(-)-beta-Pinene	0.000	0
alpha-Terpinene	0.000	0
gamma-Terpinene	0.000	0
Terpinolene	0.000	0
	**0.113%**	**1.13**

NOTES:

0

FINAL APPROVAL

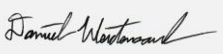

Daniel Weidensaul
20-Sep-2019
10:53 AM

PREPARED BY / DATE

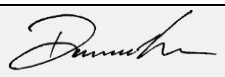

David Green
20-Sep-2019
11:17 AM

APPROVED BY / DATE

Testing results are based solely upon the sample submitted to Botanacor Laboratories, LLC. Botanacor Laboratories, LLC warrants that all analytical work is conducted professionally in accordance with all applicable standard laboratory practices using validated methods. Data was generated using an unbroken chain of comparison to NIST traceable Reference Standards and Certified Reference Materials. This report may not be reproduced, except in full, without the written approval of Botanacor Laboratories, LLC. ISO/IEC 17025:2005 Accredited A2LA Certificate Number 4329.02

botanacor

CERTIFICATE OF ANALYSIS

prepared for: ROSEMAN UNIVERSITY
10530 DISCOVERY DRIVE
LAS VEGAS, NV 89135

V4

**Batch ID:**	OHSO-ETH	**Test ID:**	7708631.0052
**Reported:**	24-Sep-2019	**Method:**	TM14
**Type:**	Concentrate		
**Test:**	Potency		

CANNABINOID PROFILE

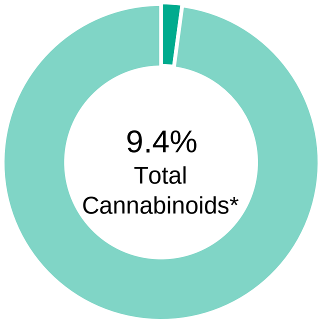

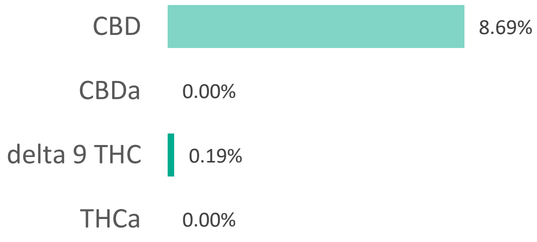

Compound	LOQ (%)	Result (%)	Result (mg/g)
Delta 9-Tetrahydrocannabinolic acid (THCA-A)	0.07	0.00	0.0
Delta 9-Tetrahydrocannabinol (Delta 9THC)	0.04	0.19	1.9
Cannabidiolic acid (CBDA)	0.07	0.00	0.0
Cannabidiol (CBD)	0.04	8.69	86.9
Delta 8-Tetrahydrocannabinol (Delta 8THC)	0.04	0.00	0.0
Cannabinolic Acid (CBNA)	0.10	0.00	0.0
Cannabinol (CBN)	0.04	0.06	0.6
Cannabigerolic acid (CBGA)	0.06	0.00	0.0
Cannabigerol (CBG)	0.04	0.15	1.5
Tetrahydrocannabivarinic Acid (THCVA)	0.06	0.00	0.0
Tetrahydrocannabivarin (THCV)	0.03	0.00	0.0
Cannabidivarinic Acid (CBDVA)	0.07	0.00	0.0
Cannabidivarin (CBDV)	0.04	0.06	0.6
Cannabichromenic Acid (CBCA)	0.05	0.00	0.0
Cannabichromene (CBC)	0.07	0.25	2.5
**Total Cannabinoids**		**9.40**	**94.00**
Total Potential THC**		0.19	1.90
Total Potential CBD**		8.69	86.90

NOTES:
N/A

% = % (w/w) = Percent (Weight of Analyte / Weight of Product)

* Total Cannabinoids result reflects the absolute sum of all cannabinoids detected.

** Total Potential THC/CBD is calculated using the following formulas to take into account the loss of a carboxyl group during decarboxylation step.

Total THC = THC + (THCa *(0.877)) and Total CBD = CBD + (CBDa *(0.877))

FINAL APPROVAL

PREPARED BY / DATE	APPROVED BY / DATE
Daniel Weidensaul<br>24-Sep-2019<br>3:25 PM	David Green<br>24-Sep-2019<br>4:17 PM

Testing results are based solely upon the sample submitted to Botanacor Laboratories, LLC, in the condition it was received. Botanacor Laboratories, LLC warrants that all analytical work is conducted professionally in accordance with all applicable standard laboratory practices using validated methods. Data was generated using an unbroken chain of comparison to NIST traceable Reference Standards and Certified Reference Materials. This report may not be reproduced, except in full, without the written approval of Botanacor Laboratories, LLC. ISO/IEC 17025:2005 Accredited A2LA Certificate Number 4329.02

ilac-MRA A2LA ACCREDITED
Certificate #4329.02

CERTIFICATE OF ANALYSIS

prepared for: ROSEMAN UNIVERSITY
10530 DISCOVERY DRIVE
LAS VEGAS, NV 89135

V4

**Batch ID:**	OHSO-ETH	**Test ID:**	9019026.0021
**Reported:**	20-Sep-2019	**Method:**	TM10
**Type:**	Concentrate		
**Test:**	Terpenes		

TERPENE PROFILE

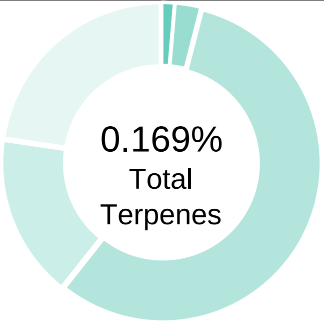

Compound	%(w/w)	mg/g
(-)-alpha-Bisabolol	0.034	0.34
Camphene	0.000	0
delta-3-Carene	0.000	0
beta-Caryophyllene	0.085	0.85
(-)-Caryophyllene Oxide	0.000	0
p-Cymene	0.000	0
Eucalyptol	0.000	0
Geraniol	0.000	0
alpha-Humulene	0.025	0.25
(-)-Isopulegol	0.000	0
d-Limonene	0.000	0
Linalool	0.004	0.04
beta-Myrcene	0.000	0
cis-Nerolidol	0.000	0
trans-Nerolidol	0.017	0.17
Ocimene	0.000	0
beta-Ocimene	0.000	0
alpha-Pinene	0.000	0
(-)-beta-Pinene	0.000	0
alpha-Terpinene	0.002	0.02
gamma-Terpinene	0.001	0.01
Terpinolene	0.001	0.01
	**0.169%**	**1.69**

PREDOMINANT TERPENES

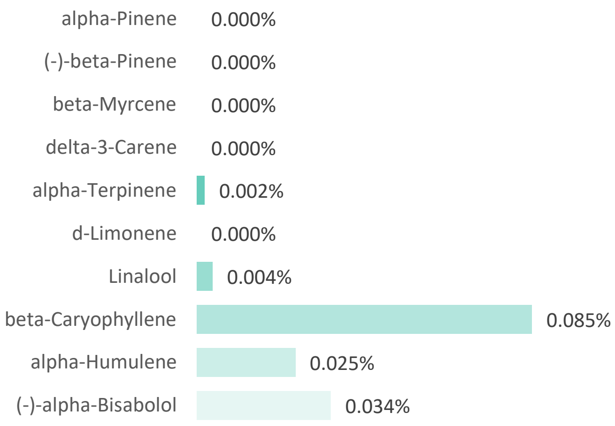

NOTES:
0

FINAL APPROVAL

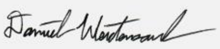
Daniel Weidensaul
20-Sep-2019
10:53 AM

PREPARED BY / DATE

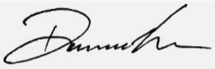
David Green
20-Sep-2019
11:17 AM

APPROVED BY / DATE

Testing results are based solely upon the sample submitted to Botanacor Laboratories, LLC. Botanacor Laboratories, LLC warrants that all analytical work is conducted professionally in accordance with all applicable standard laboratory practices using validated methods. Data was generated using an unbroken chain of comparison to NIST traceable Reference Standards and Certified Reference Materials. This report may not be reproduced, except in full, without the written approval of Botanacor Laboratories, LLC. ISO/IEC 17025:2005 Accredited A2LA Certificate Number 4329.02

CERTIFICATE OF ANALYSIS

prepared for: ROSEMAN UNIVERSITY
10530 DISCOVERY DRIVE
LAS VEGAS, NV 89135

V5

**Batch ID:**	OEVOO-ETH	**Test ID:**	7708631.0056
**Reported:**	24-Sep-2019	**Method:**	TM14
**Type:**	Concentrate		
**Test:**	Potency		

CANNABINOID PROFILE

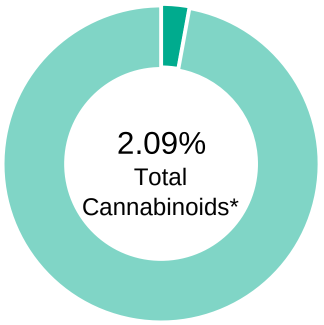

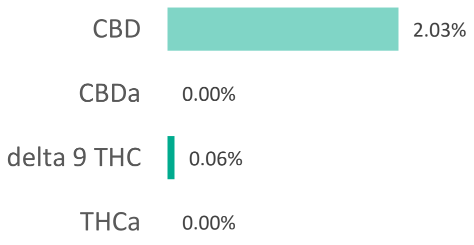

Compound	LOQ (%)	Result (%)	Result (mg/g)
Delta 9-Tetrahydrocannabinolic acid (THCA-A)	0.07	0.00	0.0
Delta 9-Tetrahydrocannabinol (Delta 9THC)	0.04	0.06	0.6
Cannabidiolic acid (CBDA)	0.07	0.00	0.0
Cannabidiol (CBD)	0.04	2.03	20.3
Delta 8-Tetrahydrocannabinol (Delta 8THC)	0.04	0.00	0.0
Cannabinolic Acid (CBNA)	0.10	0.00	0.0
Cannabinol (CBN)	0.04	0.00	0.0
Cannabigerolic acid (CBGA)	0.06	0.00	0.0
Cannabigerol (CBG)	0.04	0.00	0.0
Tetrahydrocannabivarinic Acid (THCVA)	0.06	0.00	0.0
Tetrahydrocannabivarin (THCV)	0.03	0.00	0.0
Cannabidivarinic Acid (CBDVA)	0.07	0.00	0.0
Cannabidivarin (CBDV)	0.04	0.00	0.0
Cannabichromenic Acid (CBCA)	0.06	0.00	0.0
Cannabichromene (CBC)	0.07	0.00	0.0
**Total Cannabinoids**		**2.09**	**20.90**
Total Potential THC**		0.06	0.60
Total Potential CBD**		2.03	20.30

% = % (w/w) = Percent (Weight of Analyte / Weight of Product)

* Total Cannabinoids result reflects the absolute sum of all cannabinoids detected.

** Total Potential THC/CBD is calculated using the following formulas to take into account the loss of a carboxyl group during decarboxylation step.

Total THC = THC + (THCa *(0.877)) and Total CBD = CBD + (CBDa *(0.877))

NOTES:

N/A

FINAL APPROVAL

Daniel Weidensaul
24-Sep-2019
3:25 PM

PREPARED BY / DATE

David Green
24-Sep-2019
4:17 PM

APPROVED BY / DATE

Testing results are based solely upon the sample submitted to Botanacor Laboratories, LLC, in the condition it was received. Botanacor Laboratories, LLC warrants that all analytical work is conducted professionally in accordance with all applicable standard laboratory practices using validated methods. Data was generated using an unbroken chain of comparison to NIST traceable Reference Standards and Certified Reference Materials. This report may not be reproduced, except in full, without the written approval of Botanacor Laboratories, LLC. ISO/IEC 17025:2005 Accredited A2LA Certificate Number 4329.02

Certificate #4329.02

CERTIFICATE OF ANALYSIS

prepared for: ROSEMAN UNIVERSITY
10530 DISCOVERY DRIVE
LAS VEGAS, NV 89135

V5

**Batch ID:**	OEVOO-ETH	**Test ID:**	9019026.0025
**Reported:**	20-Sep-2019	**Method:**	TM10
**Type:**	Concentrate		
**Test:**	Terpenes		

TERPENE PROFILE

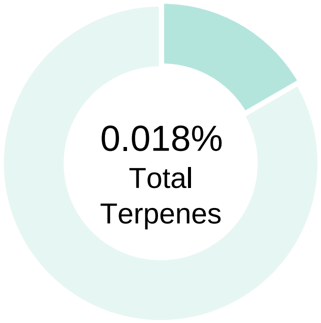

PREDOMINANT TERPENES

alpha-Pinene	0.000%
(-)-beta-Pinene	0.000%
beta-Myrcene	0.000%
delta-3-Carene	0.000%
alpha-Terpinene	0.000%
d-Limonene	0.000%
Linalool	0.000%
beta-Caryophyllene	0.003%
alpha-Humulene	0.000%
(-)-alpha-Bisabolol	0.015%

Compound	%(w/w)	mg/g
(-)-alpha-Bisabolol	0.015	0.15
Camphene	0.000	0
delta-3-Carene	0.000	0
beta-Caryophyllene	0.003	0.03
(-)-Caryophyllene Oxide	0.000	0
p-Cymene	0.000	0
Eucalyptol	0.000	0
Geraniol	0.000	0
alpha-Humulene	0.000	0
(-)-Isopulegol	0.000	0
d-Limonene	0.000	0
Linalool	0.000	0
beta-Myrcene	0.000	0
cis-Nerolidol	0.000	0
trans-Nerolidol	0.000	0
Ocimene	0.000	0
beta-Ocimene	0.000	0
alpha-Pinene	0.000	0
(-)-beta-Pinene	0.000	0
alpha-Terpinene	0.000	0
gamma-Terpinene	0.000	0
Terpinolene	0.000	0
	**0.018%**	**0.18**

NOTES:
0

FINAL APPROVAL

Daniel Weidensaul<br>20-Sep-2019<br>10:53 AM	David Green<br>20-Sep-2019<br>11:17 AM
PREPARED BY / DATE	APPROVED BY / DATE

Testing results are based solely upon the sample submitted to Botanacor Laboratories, LLC. Botanacor Laboratories, LLC warrants that all analytical work is conducted professionally in accordance with all applicable standard laboratory practices using validated methods. Data was generated using an unbroken chain of comparison to NIST traceable Reference Standards and Certified Reference Materials. This report may not be reproduced, except in full, without the written approval of Botanacor Laboratories, LLC. ISO/IEC 17025:2005 Accredited A2LA Certificate Number 4329.02

botanacor

CERTIFICATE OF ANALYSIS

prepared for: ROSEMAN UNIVERSITY
10530 DISCOVERY DRIVE
LAS VEGAS, NV 89135

V6

**Batch ID:**	MCT-ETH	**Test ID:**	7708631.0054
**Reported:**	24-Sep-2019	**Method:**	TM14
**Type:**	Concentrate		
**Test:**	Potency		

CANNABINOID PROFILE

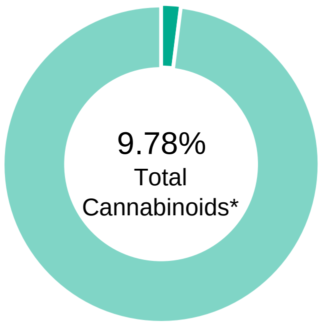

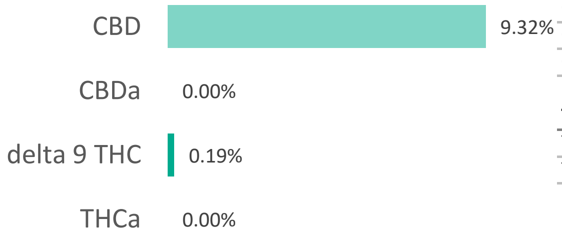

Compound	LOQ (%)	Result (%)	Result (mg/g)
Delta 9-Tetrahydrocannabinolic acid (THCA-A)	0.07	0.00	0.0
Delta 9-Tetrahydrocannabinol (Delta 9THC)	0.04	0.19	1.9
Cannabidiolic acid (CBDA)	0.07	0.00	0.0
Cannabidiol (CBD)	0.04	9.32	93.2
Delta 8-Tetrahydrocannabinol (Delta 8THC)	0.04	0.00	0.0
Cannabinolic Acid (CBNA)	0.10	0.00	0.0
Cannabinol (CBN)	0.04	0.00	0.0
Cannabigerolic acid (CBGA)	0.06	0.00	0.0
Cannabigerol (CBG)	0.04	0.00	0.0
Tetrahydrocannabivarinic Acid (THCVA)	0.06	0.00	0.0
Tetrahydrocannabivarin (THCV)	0.03	0.00	0.0
Cannabidivarinic Acid (CBDVA)	0.07	0.00	0.0
Cannabidivarin (CBDV)	0.04	0.05	0.5
Cannabichromenic Acid (CBCA)	0.05	0.00	0.0
Cannabichromene (CBC)	0.06	0.22	2.2
**Total Cannabinoids**		**9.78**	**97.80**
Total Potential THC**		0.19	1.90
Total Potential CBD**		9.32	93.20

% = % (w/w) = Percent (Weight of Analyte / Weight of Product)

* Total Cannabinoids result reflects the absolute sum of all cannabinoids detected.

** Total Potential THC/CBD is calculated using the following formulas to take into account the loss of a carboxyl group during decarboxylation step.

Total THC = THC + (THCa *(0.877)) and Total CBD = CBD + (CBDa *(0.877))

NOTES:
N/A

FINAL APPROVAL

Daniel Weidensaul
24-Sep-2019
3:25 PM

PREPARED BY / DATE

David Green
24-Sep-2019
4:17 PM

APPROVED BY / DATE

Testing results are based solely upon the sample submitted to Botanacor Laboratories, LLC, in the condition it was received. Botanacor Laboratories, LLC warrants that all analytical work is conducted professionally in accordance with all applicable standard laboratory practices using validated methods. Data was generated using an unbroken chain of comparison to NIST traceable Reference Standards and Certified Reference Materials. This report may not be reproduced, except in full, without the written approval of Botanacor Laboratories, LLC. ISO/IEC 17025:2005 Accredited A2LA Certificate Number 4329.02

ilac-MRA A2LA ACCREDITED
Certificate #4329.02

botanacor

CERTIFICATE OF ANALYSIS

prepared for: ROSEMAN UNIVERSITY
10530 DISCOVERY DRIVE
LAS VEGAS, NV 89135

V6

**Batch ID:**	MCT-ETH	**Test ID:**	9019026.0023
**Reported:**	20-Sep-2019	**Method:**	TM10
**Type:**	Concentrate		
**Test:**	Terpenes		

TERPENE PROFILE

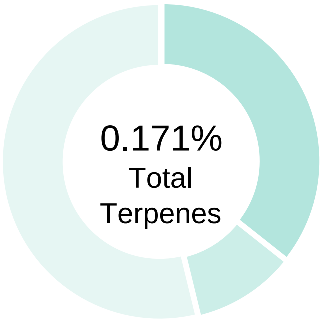

PREDOMINANT TERPENES

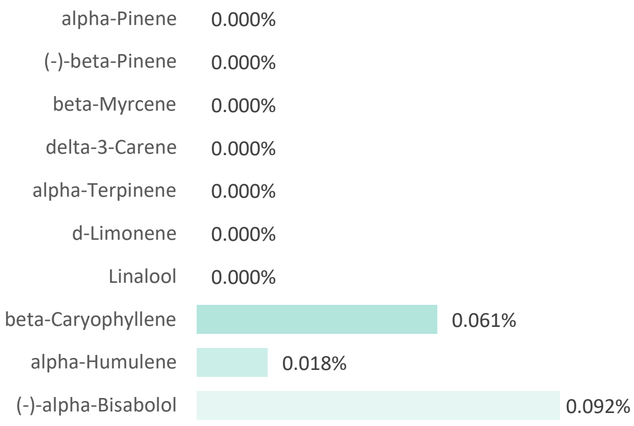

Compound	%(w/w)	mg/g
(-)-alpha-Bisabolol	0.092	0.92
Camphene	0.000	0
delta-3-Carene	0.000	0
beta-Caryophyllene	0.061	0.61
(-)-Caryophyllene Oxide	0.000	0
p-Cymene	0.000	0
Eucalyptol	0.000	0
Geraniol	0.000	0
alpha-Humulene	0.018	0.18
(-)-Isopulegol	0.000	0
d-Limonene	0.000	0
Linalool	0.000	0
beta-Myrcene	0.000	0
cis-Nerolidol	0.000	0
trans-Nerolidol	0.000	0
Ocimene	0.000	0
beta-Ocimene	0.000	0
alpha-Pinene	0.000	0
(-)-beta-Pinene	0.000	0
alpha-Terpinene	0.000	0
gamma-Terpinene	0.000	0
Terpinolene	0.000	0
	**0.171%**	**1.71**

NOTES:
0

FINAL APPROVAL

Daniel Weidensaul
20-Sep-2019
10:53 AM

PREPARED BY / DATE

David Green
20-Sep-2019
11:17 AM

APPROVED BY / DATE

Testing results are based solely upon the sample submitted to Botanacor Laboratories, LLC. Botanacor Laboratories, LLC warrants that all analytical work is conducted professionally in accordance with all applicable standard laboratory practices using validated methods. Data was generated using an unbroken chain of comparison to NIST traceable Reference Standards and Certified Reference Materials. This report may not be reproduced, except in full, without the written approval of Botanacor Laboratories, LLC. ISO/IEC 17025:2005 Accredited A2LA Certificate Number 4329.02

LABORATORY REPORT

SAMPLE NAME: V1

COMPANY NAME: Roseman University

COMPANY LOT #: NA

Column: ZB5 (60 m length × 0.25 mm inner diameter × 0.25 µm film thickness)

Instrument: Shimadzu GCMS-QP2010 Ultra

Carrier gas: Helium 80 psi

Temperature ramp: 2 degrees Celsius per minute up to 260-degree Celsius

Split ratio: 30:1

Sample preparation: 5% w/v solution with Dichloromethane

Interpretation on sample:

The analysis of this sample meets its expected terpene profile.

Analyzed by: Dr. Prabodh Satyal

Reviewed by: Ambika Poudel

Issued Date: 09/25/2019

Copyright © 2019 by Aromatic Plant Research Center (APRC). All rights reserved. The information contained in this document may not be used, published or redistributed, including online, without the prior written consent of APRC.

Sample Information

Analyzed by	: Dr. Prabodh Satyal
Analyzed	: 9/25/2019 2:36:44 AM
Sample Type	: Essential Oil
Sample Name	: V1
Company Name	: Roseman University
Lot#	: NA
Injection Volume	: 0.30

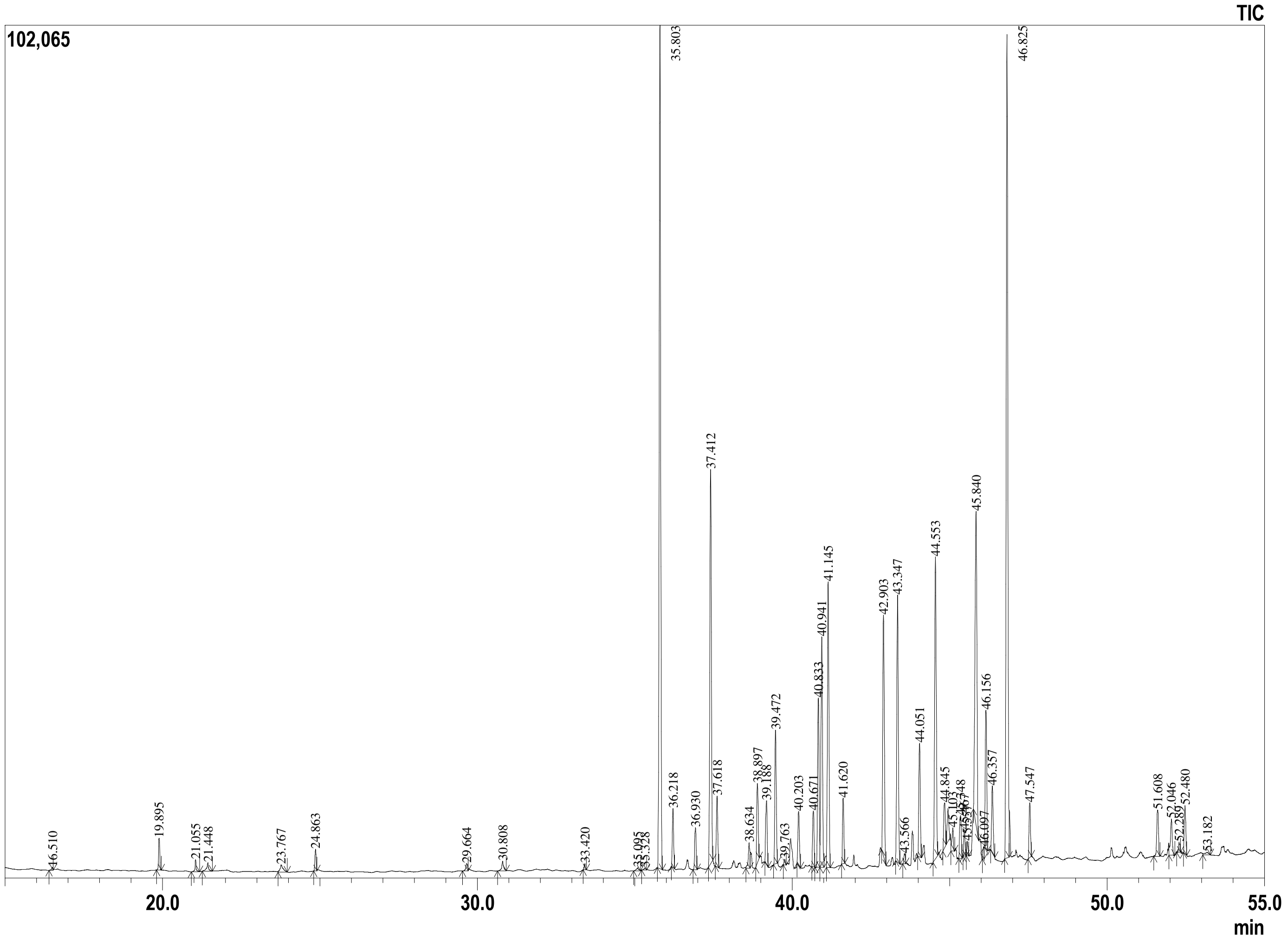

Peak Report TIC

R.Time	Name	Area%
16.510	Limonene	0.05
19.895	Linalool	0.49
21.055	Fenchol <endo>	0.19
21.448	Pinene hydrate<trans->	0.19
23.767	Borneol	0.26
24.863	Terpineol <alpha>	0.33
29.664	Decadienal<2E,4Z->	0.16
30.808	Decadienal<2E,4E->	0.23
33.420	Ylangene-alpha	0.07
35.095	Caryophyllene<cis>	0.08
35.328	Bergamotene <cis-alpha>	0.04
35.803	Caryophyllene <beta>	15.77
36.218	Bergamotene <trans-alpha>	0.95
36.930	Farnesene<(E)-beta->	0.71
37.412	Humulene <alpha>	7.25
37.618	Alloaromadendrene	1.25
38.634	Selina-4,11-diene	0.47
38.897	Selinene <beta->	1.42
39.188	Selinene <alpha>	1.11
39.472	Bisabolene <beta->	2.34
39.763	Sesquicineole	0.08
40.203	Guaine <beta>	1.00
40.671	Selinene <isomer>	0.95
40.833	Bisabolene <trans-alpha>	3.01
40.941	Guaia-3,9-diene	4.37
41.145	Selina-3,7(11)-diene	5.15
41.620	Nerolidol <trans>	1.09
42.903	Caryophyllene oxide	4.51
43.347	Guaiol	4.83
43.566	Bisabol-11-ol <cis->	0.16
44.051	Humulene epoxide II	2.06
44.553	Eudesmol <10-epi-gamma->	5.96
44.845	Eudesmol <gamma->	0.85
45.103	Caryophylla-4(12),8(13)-dien-5 beta-ol	0.38
45.348	Hedycariol	0.83
45.467	Sesquiterpene A2	0.41
45.557	Selina-3,11-dien-6-alpha-ol	0.20
45.840	Eudesmol <alpha>	7.30
46.097	Eudesmol <7-epi-alpha>	0.19
46.156	Bulnesol	2.48
46.357	Caryophyllene <14-hydroxy-9epi-E>	1.20
46.825	Bisabolol <alpha->	15.79
47.547	Juniper camphor	0.98
51.608	Cryptomeridiol <epi->	0.97
52.046	Cryptomeridiol	0.78
52.289	Neophytadiene	0.14
52.480	Phytone	0.83
53.182	Phytadiene <isomer>	0.12
		100.00

Copyright © 2019 by Aromatic Plant Research Center (APRC). All rights reserved.
The information contained in this document may not be used, published or redistributed, including online, without the prior written consent of APRC.

Reviewed by: Ambika Poudel

Hemp Analysis Report

Cannabinoid Profile Certificate of Analysis

Client:	Roseman University	Date Received:	9-20-2019
Sample Name:	V1	Date Tested:	9-23-2019
Sample Matrix:	Hemp Concentrate	APRC #:	RU190920A
Sample Lot:	N/A		

ID#	Cannabinoid	Ret. Time	Conc. (µg/mL)	% (w/w)	mg/g
1	Cannabidivarin (CBDV)	2.334	11.230	0.08	0.76
2	Cannabidiolic acid (CBDA)	2.902	4.329	0.03	0.29
3	Cannabigerolic acid (CBGA)	<LOQ	<LOQ	N/A	N/A
4	Cannabigerol (CBG)	3.260	15.011	0.10	1.02
5	Cannabidiol (CBD)	3.438	844.500	5.74	57.45
6	Tetrahydrocannabivarin (THCV)	INT	INT	N/A	N/A
7	Cannabinol (CBN)	5.059	2.597	0.02	0.18
8	Δ9-Tetrahydrocannabidinol (Δ9-THC)	6.341	21.168	0.14	1.44
9	Δ8-Tetrahydrocannabidinol (Δ8-THC)	<LOQ	<LOQ	N/A	N/A
10	Cannabichromene (CBC)	7.944	30.137	0.21	2.05
11	Δ9-Tetrahydrocannabidinolic acid (THCA-A)	<LOQ	<LOQ	N/A	N/A

	%	mg/g
Total Cannabinoids	6.32	63.20
Total THC†	0.14	1.44
Total CBD‡	5.77	57.71

*† Total THC is calculated by Δ9-THC + Δ8-THC + (THCA-A*0.877)*

*‡ Total CBD is calculated by CBD + (CBDA*0.877)*

Analyzed by: A. Anderson

Reviewed by: Dr. Prabodh Satyal

Notes: THCV could not be calculated due to interfering substances.

Copyright © 2018 by Aromatic Plant Research Center (APRC). All rights reserved.
The information contained in this document may not be used, published or redistributed, including online, without the prior written consent of APRC.

LABORATORY REPORT

SAMPLE NAME: V2

COMPANY NAME: Roseman University

COMPANY LOT #: NA

Column: ZB5 (60 m length × 0.25 mm inner diameter × 0.25 µm film thickness)

Instrument: Shimadzu GCMS-QP2010 Ultra

Carrier gas: Helium 80 psi

Temperature ramp: 2 degrees Celsius per minute up to 260-degree Celsius

Split ratio: 30:1

Sample preparation: 5% w/v solution with Dichloromethane

Interpretation on sample:

The analysis of this sample meets its expected terpene profile.

Analyzed by: Dr. Prabodh Satyal

Reviewed by: Ambika Poudel

Issued Date: 09/25/2019

Copyright © 2019 by Aromatic Plant Research Center (APRC). All rights reserved. The information contained in this document may not be used, published or redistributed, including online, without the prior written consent of APRC.

Sample Information

**Analyzed by**	**: Dr. Prabodh Satyal**
**Analyzed**	**: 9/25/2019 7:07:13 AM**
**Sample Type**	**: Essential Oil**
**Sample Name**	**: V2**
**Company Name**	**: Roseman University**
**Lot#**	**: NA**
**Injection Volume**	**: 0.30**

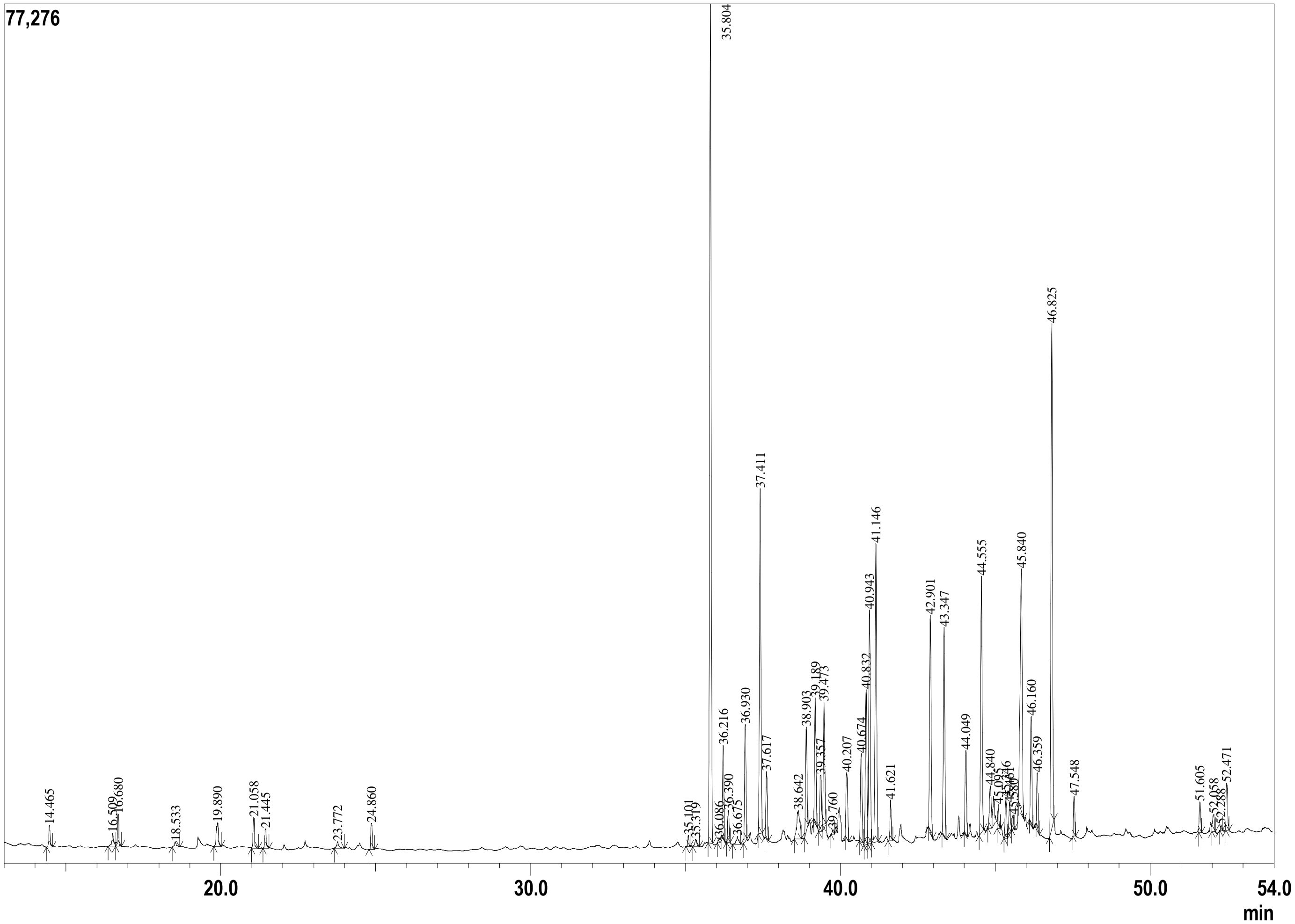

Peak Report TIC

R.Time	Name	Area%
14.465	Myrcene	0.35
16.509	Limonene	0.32
16.680	1,8-cineole	0.58
18.533	Sabinene hydrate <cis>	0.13
19.890	Linalool	0.63
21.058	Fenchol <endo>	0.56
21.445	Pinene hydrate<trans->	0.40
23.772	Borneol	0.18
24.860	Terpineol <alpha>	0.50
35.101	Caryophyllene<cis>	0.17
35.319	Bergamotene <cis-alpha>	0.21
35.804	Caryophyllene <beta>	16.89
36.086	Elemene<gamma->	0.11
36.216	Bergamotene<alpha-trans->	1.70
36.390	Guaiene <alpha>	0.58
36.675	Farnesene<Z-beta.>	0.25
36.930	Farnesene <(E)-, beta->	2.22
37.411	Humulene <alpha->	6.66
37.617	Alloaromandendrene	1.18
38.642	Selina-4,11-diene	1.13
38.903	Selinene <beta->	1.91
39.189	Selinene <alpha>	2.27
39.357	Bulnesene <alpha>	1.20
39.473	Bisabolene <beta->	2.19
39.760	Sesquicineole	0.09
40.207	Sesquiphellandrene <beta>	1.44
40.674	Selinene <isomer>	1.76
40.832	Bisabolene <trans-alpha>	3.08
40.943	Guaia-3,9-diene	4.80
41.146	Selina-3,7(11)-diene	6.05
41.621	Nerolidol <trans>	0.62
42.901	Caryophyllene oxide	4.26
43.347	Guaiol	4.08
44.049	Humulene epoxide II	1.52
44.555	Eudesmol <10-epi-gamma->	5.11
44.840	Eudesmol <gamma->	1.05
45.095	Caryophylla-4(12),8(13)-dien-5-ol	0.33
45.346	Hedycariol	0.83
45.461	Sesquiterpene A2	0.57
45.580	Selina-3,11-dien-6-alpha-ol	0.25
45.840	Eudesmol <alpha>	6.48
46.160	Bulnesol	2.03
46.359	Caryophyllene <14-hydroxy-9epi-E>	0.95
46.825	Bisabolol <alpha->	9.76
47.548	Juniper camphor	0.63
51.605	Cryptomeridiol <epi->	0.58
52.058	Cryptomeridiol	0.39
52.288	Neophytadiene	0.08
52.471	Phytone	0.94
		100.00

Copyright © 2019 by Aromatic Plant Research Center (APRC). All rights reserved.
The information contained in this document may not be used, published or redistributed, including online, without the prior written consent of APRC.

Reviewed by: Ambika Poudel

APRC®
Aromatic Plant Research Center

Hemp Analysis Report

Cannabinoid Profile Certificate of Analysis

Client:	Roseman University	Date Received:	9-20-2019
Sample Name:	V2	Date Tested:	9-23-2019
Sample Matrix:	Hemp Concentrate	APRC #:	RU190920C
Sample Lot:	N/A		

ID#	Cannabinoid	Ret. Time	Conc. (µg/mL)	% (w/w)	mg/g
1	Cannabidivarin (CBDV)	2.337	3.889	0.03	0.30
2	Cannabidiolic acid (CBDA)	2.913	17.716	0.14	1.36
3	Cannabigerolic acid (CBGA)	INT	INT	N/A	N/A
4	Cannabigerol (CBG)	3.263	6.313	0.05	0.49
5	Cannabidiol (CBD)	3.432	594.417	4.58	45.79
6	Tetrahydrocannabivarin (THCV)	INT	INT	N/A	N/A
7	Cannabinol (CBN)	5.061	1.194	0.01	0.09
8	Δ9-Tetrahydrocannabidinol (Δ9-THC)	6.343	16.287	0.13	1.25
9	Δ8-Tetrahydrocannabidinol (Δ8-THC)	6.646	0.580	0.00	0.04
10	Cannabichromene (CBC)	7.944	20.904	0.16	1.61
11	Δ9-Tetrahydrocannabidinolic acid (THCA-A)	<LOQ	<LOQ	N/A	N/A

	%	mg/g
Total Cannabinoids	5.09	50.95
Total THC†	0.13	1.30
Total CBD‡	4.70	46.99

† *Total THC is calculated by Δ9-THC + Δ8-THC + (THCA-A*0.877)*

‡ *Total CBD is calculated by CBD + (CBDA*0.877)*

Notes: CBGA, and THCV could not be calculated due to interfering substances.

Analyzed by: A. Anderson

Reviewed by: Dr. Prabodh Satyal

Copyright © 2018 by Aromatic Plant Research Center (APRC). All rights reserved.
The information contained in this document may not be used, published or redistributed, including online, without the prior written consent of APRC.

LABORATORY REPORT

SAMPLE NAME: V3

COMPANY NAME: Roseman University

COMPANY LOT #: NA

Column: ZB5 (60 m length × 0.25 mm inner diameter × 0.25 µm film thickness)

Instrument: Shimadzu GCMS-QP2010 Ultra

Carrier gas: Helium 80 psi

Temperature ramp: 2 degrees Celsius per minute up to 260-degree Celsius

Split ratio: 30:1

Sample preparation: 5% w/v solution with Dichloromethane

Interpretation on sample:

The analysis of this sample meets its expected terpene profile.

Analyzed by: Dr. Prabodh Satyal

Reviewed by: Ambika Poudel

Issued Date: 09/25/2019

Copyright © 2019 by Aromatic Plant Research Center (APRC). All rights reserved. The information contained in this document may not be used, published or redistributed, including online, without the prior written consent of APRC.

Sample Information

**Analyzed by**	**: Dr. Prabodh Satyal**
**Analyzed**	**: 9/25/2019 11:37:49 AM**
**Sample Type**	**: Essential Oil**
**Sample Name**	**: V3**
**Company Name**	**: Roseman University**
**Lot#**	**: NA**
**Injection Volume**	**: 0.30**

APRC
Aromatic Plant Research Center

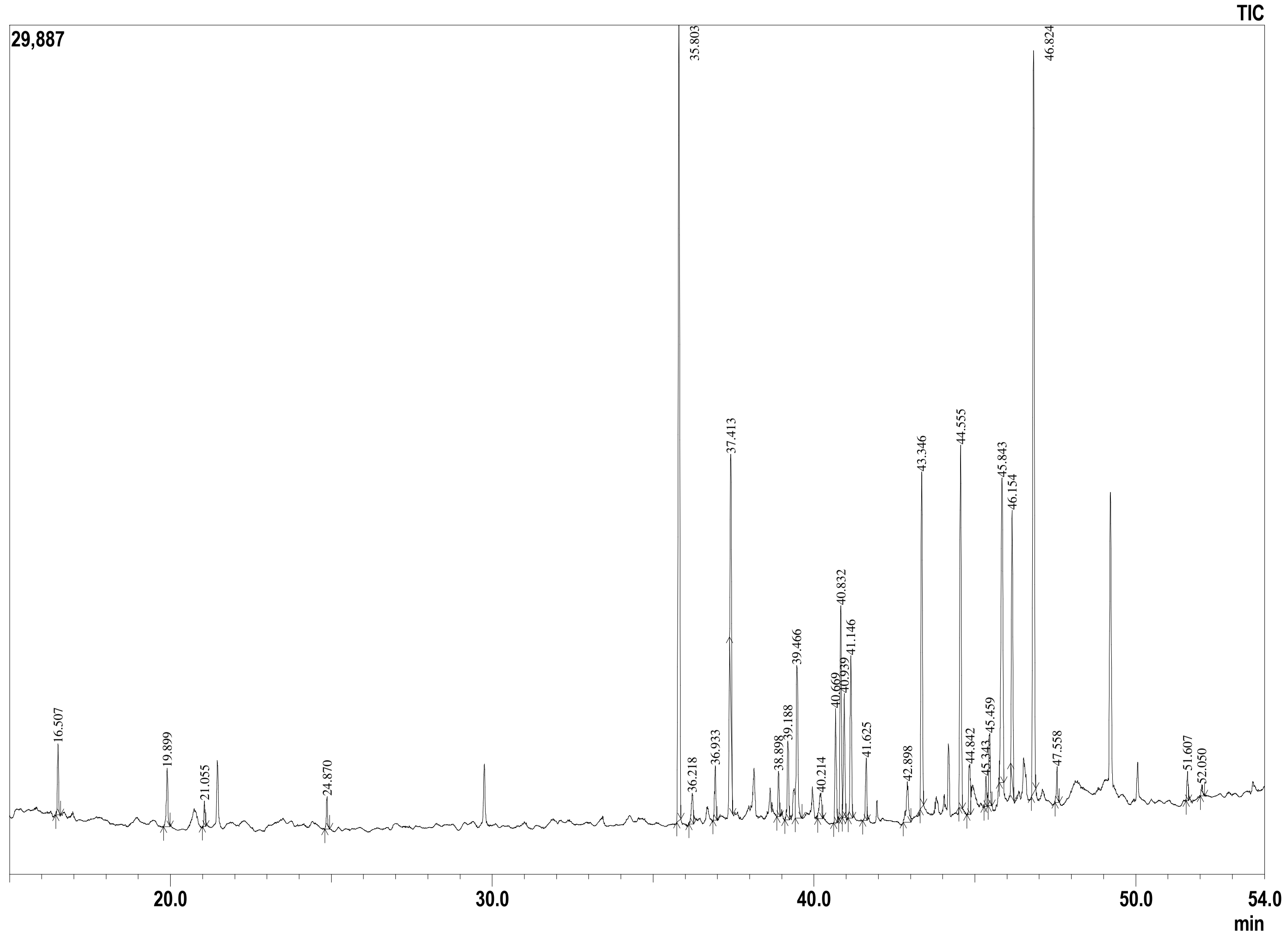

Peak Report TIC

R.Time	Name	Area%
16.507	Limonene	1.18
19.899	Linalool	1.36
21.055	Fenchol <endo>	0.39
24.870	Terpineol <alpha>	0.56
35.803	Caryophyllene <beta>	17.61
36.218	Bergamotene <trans-alpha>	0.82
36.933	Farnesene<(E)-beta->	1.07
37.413	Humulene <alpha>	4.55
38.898	Selinene <beta->	0.82
39.188	Farnesene <E,E-alpha>	1.74
39.466	Bisabolene <beta->	3.80
40.214	Sesquiphellandrene <beta>	0.82
40.669	Selinene <isomer>	2.17
40.832	Bisabolene <trans-alpha>	4.42
40.939	Guaia-3,9-diene	2.42
41.146	Selina-3,7(11)-diene	3.44
41.625	Nerolidol <trans>	1.18
42.898	Caryophyllene oxide	1.38
43.346	Guaiol	7.08
44.555	Eudesmol <10-epi-gamma->	7.87
44.842	Eudesmol <gamma>	1.21
45.343	Hedycariol	0.50
45.459	Sesquiterpene A2	1.53
45.843	Eudesmol<alpha->	8.50
46.154	Bulnesol	5.32
46.824	Bisabolol <alpha->	16.86
47.558	Juniper camphor	0.64
51.607	Cryptomeridiol <epi->	0.51
52.050	Cryptomeridiol	0.24
		100.00

Copyright © 2019 by Aromatic Plant Research Center (APRC). All rights reserved.
The information contained in this document may not be used, published or redistributed, including online, without the prior written consent of APRC.

Reviewed by: Ambika Poudel

Hemp Analysis Report

Cannabinoid Profile Certificate of Analysis

Client:	Roseman University	Date Received:	9-20-2019
Sample Name:	V3	Date Tested:	9-23-2019
Sample Matrix:	Hemp Concentrate	APRC #:	RU190920E
Sample Lot:	N/A		

ID#	Cannabinoid	Ret. Time	Conc. (µg/mL)	% (w/w)	mg/g
1	Cannabidivarin (CBDV)	2.333	3.338	0.02	0.22
2	Cannabidiolic acid (CBDA)	<LOQ	<LOQ	N/A	N/A
3	Cannabigerolic acid (CBGA)	ND	ND	N/A	N/A
4	Cannabigerol (CBG)	3.258	8.601	0.06	0.56
5	Cannabidiol (CBD)	3.427	971.500	6.34	63.37
6	Tetrahydrocannabivarin (THCV)	INT	INT	N/A	N/A
7	Cannabinol (CBN)	<LOQ	<LOQ	N/A	N/A
8	Δ9-Tetrahydrocannabidinol (Δ9-THC)	6.335	22.593	0.15	1.47
9	Δ8-Tetrahydrocannabidinol (Δ8-THC)	<LOQ	<LOQ	N/A	N/A
10	Cannabichromene (CBC)	7.936	25.438	0.17	1.66
11	Δ9-Tetrahydrocannabidinolic acid (THCA-A)	<LOQ	<LOQ	N/A	N/A

Analyzed by: A. Anderson

Reviewed by: Dr. Prabodh Satyal

	%	mg/g
Total Cannabinoids	6.73	67.28
Total THC†	0.15	1.47
Total CBD‡	6.34	63.37

*† Total THC is calculated by Δ9-THC + Δ8-THC + (THCA-A*0.877)*

*‡ Total CBD is calculated by CBD + (CBDA*0.877)*

Notes: THCV could not be calculated due to interfering substances.

Copyright © 2018 by Aromatic Plant Research Center (APRC). All rights reserved.
The information contained in this document may not be used, published or redistributed, including online, without the prior written consent of APRC.

LABORATORY REPORT

SAMPLE NAME: V4

COMPANY NAME: Roseman University

COMPANY LOT #: NA

Column: ZB5 (60 m length × 0.25 mm inner diameter × 0.25 µm film thickness)

Instrument: Shimadzu GCMS-QP2010 Ultra

Carrier gas: Helium 80 psi

Temperature ramp: 2 degrees Celsius per minute up to 260-degree Celsius

Split ratio: 30:1

Sample preparation: 5% w/v solution with Dichloromethane

Interpretation on sample:

The analysis of this sample meets its expected terpene profile.

Analyzed by: Dr. Prabodh Satyal

Reviewed by: Ambika Poudel

Issued Date: 09/25/2019

Copyright © 2019 by Aromatic Plant Research Center (APRC). All rights reserved. The information contained in this document may not be used, published or redistributed, including online, without the prior written consent of APRC.

Sample Information

**Analyzed by**	**: Dr. Prabodh Satyal**
**Analyzed**	**: 9/25/2019 4:51:58 AM**
**Sample Type**	**: Essential Oil**
**Sample Name**	**: V4**
**Company Name**	**: Roseman University**
**Lot#**	**: NA**
**Injection Volume**	**: 0.30**

APRC
Aromatic Plant Research Center

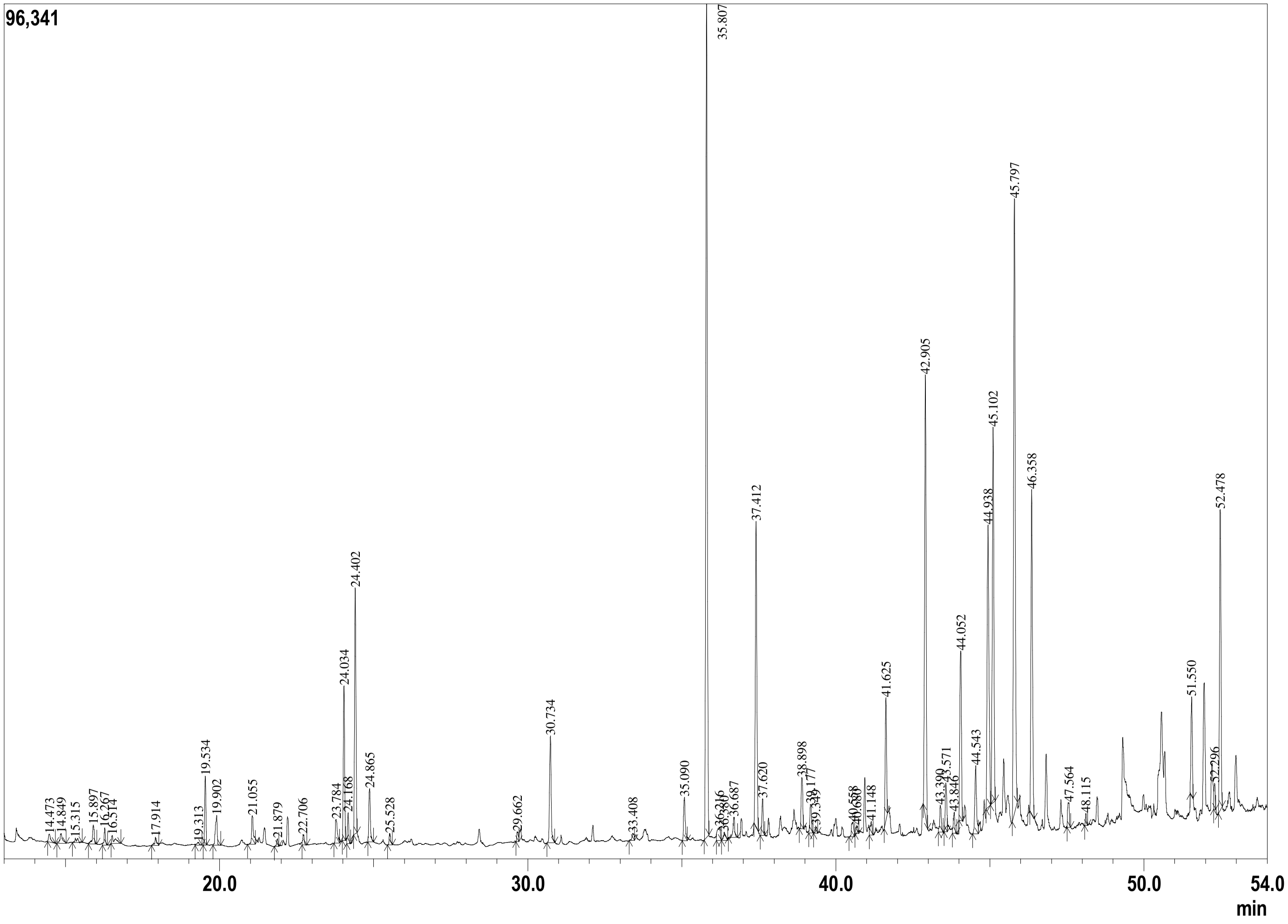

Peak Report TIC

R.Time	Name	Area%
14.473	Myrcene	0.21
14.849	Heptadienal<2E,4E->	0.32
15.315	Cycloheptane<trimethyl>	0.14
15.897	Terpinene <alpha->	0.44
16.267	Cymene <para>	0.28
16.514	Limonene	0.29
17.914	Terpinene<gamma->	0.22
19.313	Terpiniolene	0.08
19.534	Cymenene <para->	1.09
19.902	Linalool	0.65
21.055	Fenchol <endo>	0.65
21.879	Mentha-2,8-dien-1-ol <cis-para>	0.13
22.706	Pinene oxide<beta>	0.19
23.784	Borneol	0.61
24.034	1,8-menthadien-4-ol <para>	2.62
24.168	Terpinen-4-ol	0.50
24.402	Cymen-8-ol<para->	4.15
24.865	Terpineol <alpha>	0.96
25.528	Verbenone	0.22
29.662	Decadienal<2E,4Z->	0.20
30.734	Cryptone<4-hydroxy>+Terpinyl acetate<delta>	2.08
33.408	Ylangene <alpha>	0.18
35.090	Caryophyllene <cis>	0.86
35.807	Caryophyllene <beta>	15.65
36.216	Bergamotene <trans-alpha>	0.47
36.380	Aromadendrene	0.25
36.687	Geranyl acetone	0.44
37.412	Humulene <alpha>	5.84
37.620	Alloaromadendrene	0.62
38.898	Selinene <beta->	0.96
39.177	alpha-Selinene	0.56
39.349	Bulnesene <alpha>	0.12
40.558	Dihydroactinidiolide	0.40
40.680	Guaia-3,9-diene	0.16
41.148	Selina-3,7(11)-diene	0.29
41.625	Nerolidol <trans>	2.16
42.905	Caryophyllene oxide	8.31
43.390	Hydrolysed caryophyllene	0.52
43.571	Bisabol-11-ol <trans->	0.91
43.846	Ledol	0.38
44.052	Humulene epoxide II	3.26
44.543	Selina-6-en-4-ol	1.49
44.938	Spathulenol<iso->	6.10
45.102	Caryophylla-4(12),8(13)-dien-5 beta-ol	7.25
45.797	Caryophyllene <14-hydroxy-9epi-Z>	12.27
46.358	Caryophyllene <14-hydroxy-9epi-E>	6.10
47.564	Juniper camphor	0.50
48.115	Atlantone isomer	0.16
51.550	Nootkatone	1.70
52.296	Neophytadiene	0.36
52.478	Phytone	5.73
		100.00

Copyright © 2019 by Aromatic Plant Research Center (APRC). All rights reserved.
The information contained in this document may not be used, published or redistributed, including online, without the prior written consent of APRC.

Reviewed by: Ambika Poudel

Hemp Analysis Report

Cannabinoid Profile Certificate of Analysis

Client:	Roseman University	Date Received:	9-20-2019
Sample Name:	V4	Date Tested:	9-23-2019
Sample Matrix:	Hemp Concentrate	APRC #:	RU190920B
Sample Lot:	N/A		

ID#	Cannabinoid	Ret. Time	Conc. (μg/mL)	% (w/w)	mg/g
1	Cannabidivarin (CBDV)	2.327	7.550	0.07	0.65
2	Cannabidiolic acid (CBDA)	2.902	6.214	0.05	0.54
3	Cannabigerolic acid (CBGA)	INT	INT	N/A	N/A
4	Cannabigerol (CBG)	3.258	7.540	0.07	0.65
5	Cannabidiol (CBD)	3.437	863.500	7.46	74.57
6	Tetrahydrocannabivarin (THCV)	INT	INT	N/A	N/A
7	Cannabinol (CBN)	5.057	3.638	0.03	0.31
8	Δ9-Tetrahydrocannabidinol (Δ9-THC)	6.338	21.916	0.19	1.89
9	Δ8-Tetrahydrocannabidinol (Δ8-THC)	INT	INT	N/A	N/A
10	Cannabichromene (CBC)	7.939	25.123	0.22	2.17
11	Δ9-Tetrahydrocannabidinolic acid (THCA-A)	<LOQ	<LOQ	N/A	N/A

Analyzed by: A. Anderson

Reviewed by: Dr. Prabodh Satyal

	%	mg/g
Total Cannabinoids	8.08	80.78
Total THC†	0.19	1.89
Total CBD‡	7.50	75.04

*† Total THC is calculated by Δ9-THC + Δ8-THC + (THCA-A*0.877)*

*‡ Total CBD is calculated by CBD + (CBDA*0.877)*

Notes: CBGA, THCV, and Δ8-THC could not be calculated due to interfering substances. Even counting the false THC peak, total THC remains below 0.3 %

Copyright © 2018 by Aromatic Plant Research Center (APRC). All rights reserved.
The information contained in this document may not be used, published or redistributed, including online, without the prior written consent of APRC.

LABORATORY REPORT

SAMPLE NAME: V5

COMPANY NAME: Roseman University

COMPANY LOT #: NA

Column: ZB5 (60 m length × 0.25 mm inner diameter × 0.25 µm film thickness)

Instrument: Shimadzu GCMS-QP2010 Ultra

Carrier gas: Helium 80 psi

Temperature ramp: 2 degrees Celsius per minute up to 260-degree Celsius

Split ratio: 30:1

Sample preparation: 5% w/v solution with Dichloromethane

Interpretation on sample:

The analysis of this sample meets its expected terpene profile.

Analyzed by: Dr. Prabodh Satyal

Reviewed by: Ambika Poudel

Issued Date: 09/26/2019

Copyright © 2019 by Aromatic Plant Research Center (APRC). All rights reserved. The information contained in this document may not be used, published or redistributed, including online, without the prior written consent of APRC.

Sample Information

**Analyzed by**	**: Dr. Prabodh Satyal**
**Analyzed**	**: 9/25/2019 1:53:25 PM**
**Sample Type**	**: Essential Oil**
**Sample Name**	**: V5**
**Company Name**	**: Roseman University**
**Lot#**	**: NA**
**Injection Volume**	**: 0.30**

APRC
Aromatic Plant Research Center

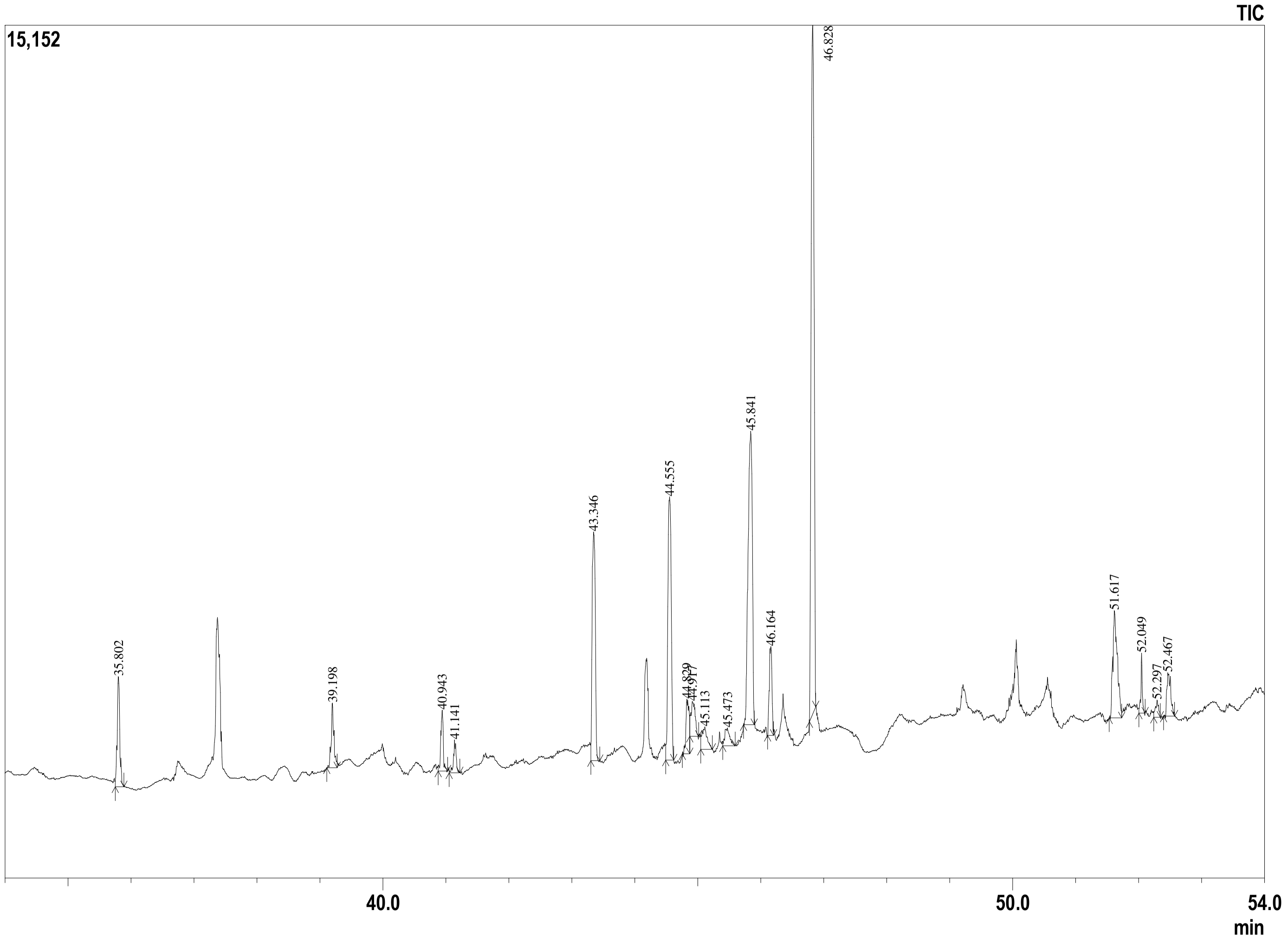

Peak Report TIC

R.Time	Name	Area%
35.802	Caryophyllene <beta>	4.17
39.198	Farnesene<(E,E)-alpha->	2.44
40.943	Guaia-3,9-diene	2.08
41.141	Selina-3,7(11)-diene	1.15
43.346	Guaiol	10.00
44.555	Eudesmol <10-epi-gamma->	12.66
44.829	Eudesmol <gamma>	2.44
44.917	Caryophylla-4(12),8(13)-dien-5 alpha-ol	2.06
45.113	Caryophylla-4(12),8(13)-dien-5 beta-ol	1.59
45.473	Sesquiterpene A2	1.21
45.841	Eudesmol <alpha>	17.51
46.164	Bulnesol	3.29
46.828	Bisabolol <alpha->	28.23
51.617	Cryptomeridiol <epi->	6.90
52.049	Cryptomeridiol	1.20
52.297	Neophytadiene	0.73
52.467	Phytone	2.34
		100.00

Copyright © 2019 by Aromatic Plant Research Center (APRC). All rights reserved.
The information contained in this document may not be used, published or redistributed, including online, without the prior written consent of APRC.

Reviewed by: Ambika Poudel

Hemp Analysis Report

Cannabinoid Profile Certificate of Analysis

Client: Roseman University
Sample Name: V5
Sample Matrix: Hemp Concentrate
Sample Lot: N/A

Date Received: 9-20-2019
Date Tested: 9-23-2019
APRC #: RU190920F

ID#	Cannabinoid	Ret. Time	Conc. (µg/mL)	% (w/w)	mg/g
1	Cannabidivarin (CBDV)	2.312	1.133	0.01	0.08
2	Cannabidiolic acid (CBDA)	2.898	3.679	0.03	0.25
3	Cannabigerolic acid (CBGA)	<LOQ	<LOQ	N/A	N/A
4	Cannabigerol (CBG)	3.247	3.841	0.03	0.26
5	Cannabidiol (CBD)	3.422	285.000	1.94	19.39
6	Tetrahydrocannabivarin (THCV)	INT	INT	N/A	N/A
7	Cannabinol (CBN)	5.040	1.433	0.01	0.10
8	Δ9-Tetrahydrocannabidinol (Δ9-THC)	6.318	9.044	0.06	0.62
9	Δ8-Tetrahydrocannabidinol (Δ8-THC)	<LOQ	<LOQ	N/A	N/A
10	Cannabichromene (CBC)	7.914	8.220	0.06	0.56
11	Δ9-Tetrahydrocannabidinolic acid (THCA-A)	<LOQ	<LOQ	N/A	N/A

Analyzed by: A. Anderson

Reviewed by: Dr. Prabodh Satyal

	%	mg/g
Total Cannabinoids	2.12	21.25
Total THC†	0.06	0.62
Total CBD‡	1.96	19.61

*† Total THC is calculated by Δ9-THC + Δ8-THC + (THCA-A*0.877)*

*‡ Total CBD is calculated by CBD + (CBDA*0.877)*

Notes: THCV could not be calculated due to interfering substances.

Copyright © 2018 by Aromatic Plant Research Center (APRC). All rights reserved.
The information contained in this document may not be used, published or redistributed, including online, without the prior written consent of APRC.

LABORATORY REPORT

SAMPLE NAME: V6

COMPANY NAME: Roseman University

COMPANY LOT #: NA

Column: ZB5 (60 m length × 0.25 mm inner diameter × 0.25 µm film thickness)

Instrument: Shimadzu GCMS-QP2010 Ultra

Carrier gas: Helium 80 psi

Temperature ramp: 2 degrees Celsius per minute up to 260-degree Celsius

Split ratio: 30:1

Sample preparation: 5% w/v solution with Dichloromethane

Interpretation on sample:

The analysis of this sample meets its expected terpene profile.

Analyzed by: Dr. Prabodh Satyal

Reviewed by: Ambika Poudel

Issued Date: 09/25/2019

Copyright © 2019 by Aromatic Plant Research Center (APRC). All rights reserved. The information contained in this document may not be used, published or redistributed, including online, without the prior written consent of APRC.

Sample Information

**Analyzed by**	**: Dr. Prabodh Satyal**
**Analyzed**	**: 9/25/2019 9:22:29 AM**
**Sample Type**	**: Essential Oil**
**Sample Name**	**: V6**
**Company Name**	**: Roseman University**
**Lot#**	**: NA**
**Injection Volume**	**: 0.30**

APRC
Aromatic Plant Research Center

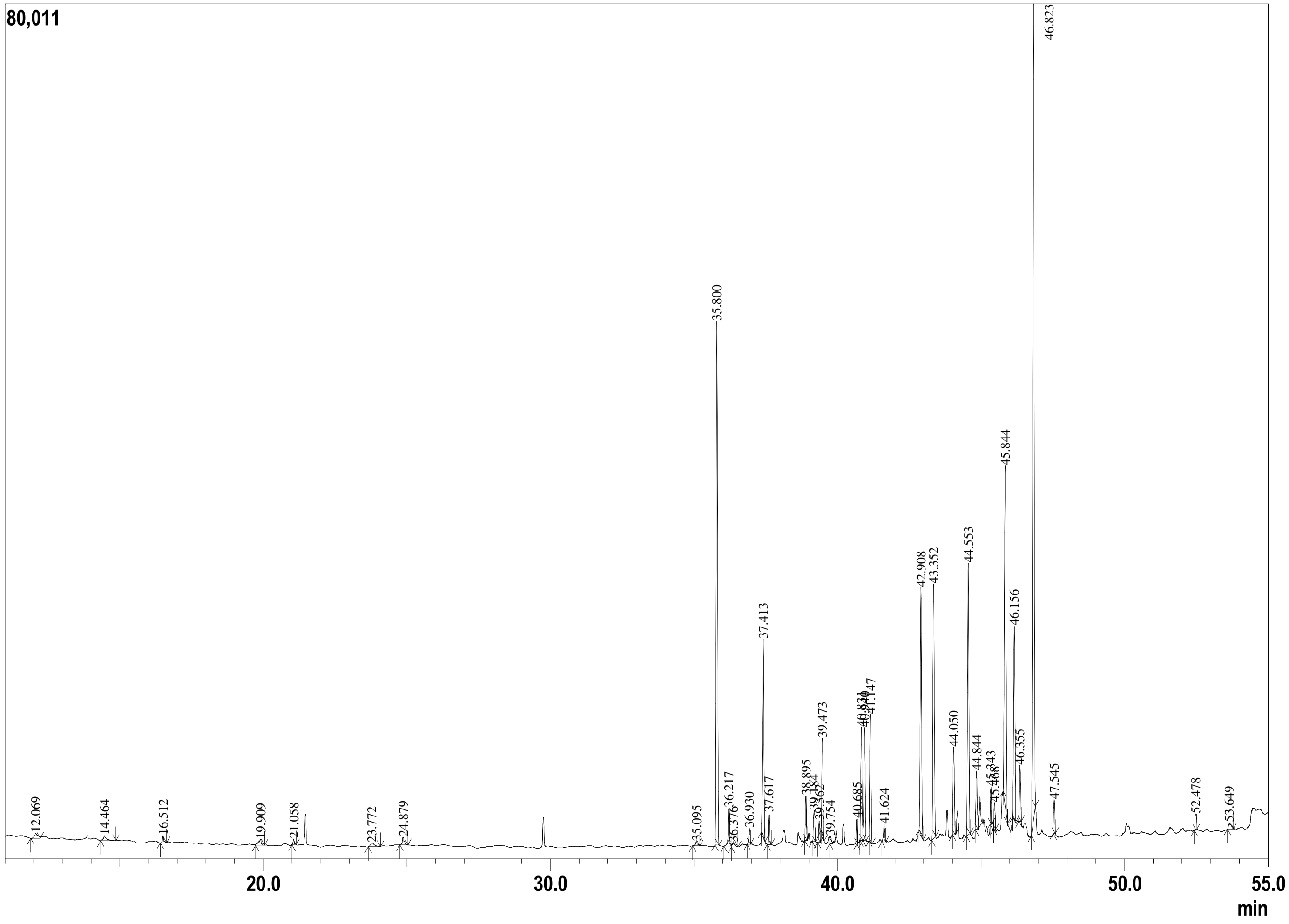

Peak Report TIC

R.Time	Name	Area%
12.069	Pinene <alpha>	0.31
14.464	Myrcene	0.30
16.512	Limonene	0.18
19.909	Linalool	0.28
21.058	Fenchol<endo->	0.14
23.772	Borneol	0.28
24.879	Terpineol <alpha>	0.29
35.095	Caryophyllene<cis>	0.16
35.800	Caryophyllene <beta>	13.36
36.217	Bergamotene <trans-alpha>	1.02
36.376	Guaine-alpha	0.19
36.930	Farnesene <E-beta>	0.34
37.413	Humulene <alpha>	5.29
37.617	Alloaromandendrene	0.76
38.895	Selinene <beta->	1.01
39.184	Selinene <alpha>	0.83
39.362	Bulnesene-alpha	0.41
39.473	Bisabolene <beta->	2.17
39.754	Sesquicineole	0.21
40.685	Sesquiphellandrene <beta>	0.25
40.831	Bisabolene <trans-alpha>	2.70
40.940	Guaia-3,9-diene	2.81
41.147	Selina-3,7(11)-diene	3.26
41.624	Nerolidol <trans>	0.38
42.908	Caryophyllene oxide	6.29
43.352	Guaiol	6.68
44.050	Humulene epoxide II	2.10
44.553	Eudesmol <10-epi-gamma->	7.12
44.844	Eudesmol<gamma->	1.48
45.343	Hedycariol	0.58
45.468	Sesquiterpene A2	0.51
45.844	Eudesmol <alpha>	10.16
46.156	Bulnesol	4.80
46.355	Caryophyllene <14-hydroxy-9epi-E>	1.27
46.823	Bisabolol <alpha->	20.66
47.545	Juniper camphor	0.78
52.478	Phytone	0.29
53.649	Phytadiene <isomer>	0.32
		100.00

Copyright © 2019 by Aromatic Plant Research Center (APRC). All rights reserved.
The information contained in this document may not be used, published or redistributed, including online, without the prior written consent of APRC.

Reviewed by: Ambika Poudel

Hemp Analysis Report

Cannabinoid Profile Certificate of Analysis

Client:	Roseman University	Date Received:	9-20-2019
Sample Name:	V6	Date Tested:	9-23-2019
Sample Matrix:	Hemp Concentrate	APRC #:	RU190920D
Sample Lot:	N/A		

ID#	Cannabinoid	Ret. Time	Conc. (µg/mL)	% (w/w)	mg/g
1	Cannabidivarin (CBDV)	2.335	6.055	0.04	0.43
2	Cannabidiolic acid (CBDA)	2.910	6.221	0.04	0.44
3	Cannabigerolic acid (CBGA)	INT	INT	N/A	N/A
4	Cannabigerol (CBG)	3.262	6.046	0.04	0.43
5	Cannabidiol (CBD)	3.439	1137.583	8.05	80.51
6	Tetrahydrocannabivarin (THCV)	INT	INT	N/A	N/A
7	Cannabinol (CBN)	5.061	1.588	0.01	0.11
8	Δ9-Tetrahydrocannabidinol (Δ9-THC)	6.342	25.634	0.18	1.81
9	Δ8-Tetrahydrocannabidinol (Δ8-THC)	6.642	2.382	0.02	0.17
10	Cannabichromene (CBC)	7.944	27.199	0.19	1.92
11	Δ9-Tetrahydrocannabidinolic acid (THCA-A)	<LOQ	<LOQ	N/A	N/A

	%	mg/g
Total Cannabinoids	8.58	85.83
Total THC†	0.20	1.98
Total CBD‡	8.09	80.89

Analyzed by: A. Anderson

Reviewed by: Dr. Prabodh Satyal

*† Total THC is calculated by Δ9-THC + Δ8-THC + (THCA-A*0.877)*

*‡ Total CBD is calculated by CBD + (CBDA*0.877)*

Notes: CBGA, and THCV could not be calculated due to interfering substances.

Copyright © 2018 by Aromatic Plant Research Center (APRC). All rights reserved.
The information contained in this document may not be used, published or redistributed, including online, without the prior written consent of APRC.

dōTERRA

Sample type: Sample
Sample name: CBD oils
Analyst: Michelle Workman
Injection date: 9/18/2019

Cannabinoid Profile

	Total CBD	Total THC
V3	6.14%	0.14%
V6	8.49%	0.20%
V1	7.15%	0.23%
V2	4.40%	0.16%
V4	6.77%	0.22%
V5	2.19%	0.06%

Analyst: [signature] Date: 9/30/19 Reviewer: [signature] Date: 9/30/19
